# Impact of *GTF2H1* and *RAD54L2* polymorphisms on the risk of lung cancer in the Chinese Han population

**DOI:** 10.1186/s12885-022-10303-1

**Published:** 2022-11-16

**Authors:** Tingting Geng, Miao Li, Rong Chen, Shuangyu Yang, Guoquan Jin, Tinabo Jin, Fulin Chen

**Affiliations:** 1 Key Laboratory of Resource Biology and Biotechnology in Western China, Ministry of Education, School of Medicine, Northwest University, #229 Taibai North Road, Xi’an, 710069 Shaanxi China; 2 Department of Medicine Oncology, The Fifth People’s Hospital of Qinghai Province, 810007 Xining, Qinghai, China; 3 Department of Medicine Oncology, The Affiliated Hospital of Qinghai University, 810016 Xining, Qinghai, China

**Keywords:** Lung cancer, Gene polymorphisms, *GTF2H1*, *RAD54L2*, MDR analysis

## Abstract

**Background:**

Repair pathway genes play an important role in the development of lung cancer. The study aimed to assess the correlation between single nucleotide polymorphisms (SNPs) in DNA repair gene (*GTF2H1* and *RAD54L2*) and the risk of lung cancer.

**Methods:**

Five SNPs in *GTF2H1* and four SNPs in *RAD54L2* in 506 patients with lung cancer and 510 age-and gender-matched healthy controls were genotyped via the Agena MassARRAY platform. The influence of *GTF2H1* and *RAD54L2* polymorphisms on lung cancer susceptibility was assessed using logistic regression analysis by calculating odds ratios (ORs) and their corresponding 95% confidence intervals (CIs).

**Results:**

*RAD54L2* rs9864693 GC genotype increased the risk of lung cancer (OR = 1.33, 95%CI: 1.01–1.77, *p* = 0.045). Stratified analysis found that associations of *RAD54L2* rs11720298, *RAD54L2* rs4687592, *RAD54L2* rs9864693 and *GTF2H1* rs4150667 with lung cancer risk were found in subjects aged ≤ 59 years. Precisely, a protective effect of *RAD54L2* rs11720298 on the occurrence of lung cancer was observed in non-smokers and drinkers. *GTF2H1* rs4150667 was associated with a decreased risk of lung cancer in subjects with BMI ≤ 24 kg/m^2^. *RAD54L2* rs4687592 was associated with an increased risk of lung cancer in drinkers. In addition, *GTF2H1* rs3802967 was associated with a reduced risk of lung squamous cell carcinoma.

**Conclusion:**

Our study first revealed that *RAD54L2* rs9864693 was associated with an increased risk of lung cancer in the Chinese Han population. This study may increase the understanding of the effect of *RAD54L2* and *GTF2H1* polymorphisms on lung cancer occurrence.

**Supplementary Information:**

The online version contains supplementary material available at 10.1186/s12885-022-10303-1.

## Introduction

Lung cancer, also called primary bronchogenic carcinoma, is a malignant tumor derived from the trachea, bronchial mucosa or glands. Currently, it is the most common malignant tumor in the world [1]. According to statistics, in 2015, lung cancer is the most important cause of death in cancer patients, and the incidences of lung cancer in Chinese men and women were 50.9 per 100,000 person-years and 22.4 per 100,000 person-years, respectively [2]. Surgery is still the preferred treatment for early-stage lung cancer. However, most lung cancer patients are diagnosed at an advanced stage, and their 5-year survival rate is only 19.7%, that is to say, they missed the best treatment opportunity [1, 3]. Most studies have suggested that the occurrence of lung cancer is related to environmental factors, such as smoking, occupational exposure, and air pollution [4, 5]. At the same, genetic factors play an important role in the development of lung cancer, including *EGFR* [6], *CHRNA5* [7], *CLPTM1L* [8], *TP63* [9], and so on.

*GTF2H1* (general transcription factor IIH subunit 1) protein plays a vital role in the nucleotide excision repair (NER) pathway, participates in the early damage recognition of XPC-HR23B protein, and recruits endonuclease XPG to the injury site to complete the enzymatic cleavage process [10–12]. In addition, GTF2H1 protein is involved in transcription and regulates the transcriptional activation of multiple genes [13]. One research has found that rs3802967 and rs4150606 in the *GTF2H1* gene may increase the risk of lung cancer, and rs4150667 in the *GTF2H1* gene variant may reduce the risk of lung cancer [14]. So, the genetic polymorphisms of *GTF2H1* may be involved in the pathogenesis of lung cancer.

*RAD54L2*, also known as *ARIP4* (androgen receptor-interacting protein 4), is a protein-coding gene belonging to the RAD54 subfamily of SNF2-type chromatin remodeling factor superfamily [15], with double-stranded DNA-dependent ATPase activity. In fact, Rad54 interacts with Rad51, thereby enhancing its ability to form cruciform and D-loops. *RAD51* catalyzes DNA repair by homologous recombination to ensure the stability of cell genome. In a study of the effects of *RAD51* G135C polymorphism on non-small cell lung cancer patients treated with platinum-paclitaxel/gemcitabine Wrst line chemotherapy, it has been found that the G135C allele is associated with a higher survival time and a better prognosis [16]. However, the effect of the *RAD54L2* gene on the occurrence and development of lung cancer is unknown.

Here, nine polymorphisms in *GTF2H1* and *RAD54L2* were selected and genotyped to explore their impact on the risk of lung cancer in the Chinese Han population.

## Materials and methods

### Study participants

A total of 506 patients with lung cancer and 510 age-and gender-matched healthy controls were recruited from the Qinghai Province Cancer Hospital. The inclusion criteria for cases were lung cancer confirmed by histopathology and no history of malignant tumors in other organs. Patients with prior cancer history, pulmonary diseases, and serious chronic diseases were excluded. The control group consisted of healthy people with no medical or family history of cancer or any lung disease. Further, each subject was interviewed by trained personnel using a structured questionnaire to obtain information about demographic characteristics [age, gender, smoking and drinking, body mass index (BMI)]. Based on smoking status, participants were classified into non-smokers (never) and smokers (ever or current smokers). Subjects who smoked at least one cigarette per day were classified as current smokers. For drinking status, participants were classified into non-drinkers (never) and drinkers (ever or current alcohol drinkers). Subjects who drank at least 100 g of alcohol per week were considered as drinkers. Pathological data (pathological type, lymph node metastasis, and clinical stage) were obtained via medical records. This study was conducted under the approval of the Institutional Review Boards of Northwest University. All participants were informed of the content of the study and signed informed consent. It was confirmed that all methods were carried out in accordance with relevant guidelines and regulations.

### SNP selection and genotyping

The GoldMag-Mini Whole Blood Genomic DNA Purification Kit (GoldMag Co. Ltd. Xi’an City, China) was applied to extract DNA samples from the 5 mL peripheral venous blood, and Nanodrop 2000 (Gene Company Limited) was used to detect the concentration and purity of DNA samples to ensure that samples could be used for subsequent experiments. Four SNPs (rs11720298, rs4687721, rs4687592, and rs9864693) in *RAD54L2* and five SNPs (rs4150530, rs3802967, rs4150606, rs4150658, and rs4150667) in GTF2H1 were randomly selected in our study based on the following criteria: (1) minor allele frequency (MAF) > 0.05, min genotype > 75%, and r^2^ > 0.8 from the Genome Aggregation Database (gnomAD, http://www.gnomad-sg.org/), (2) MAF more than 0.05 in the Chinese Han population from dbSNP database (https://www.ncbi.nlm.nih.gov/snp/), (3) previous studies on these polymorphisms have been related to lung cancer [14], (4) combined with MassARRAY primer design software, Hardy-Weinberg equilibrium (HWE) > 0.05 and the call rate > 95% in our study population. Haploreg (https://pubs.broadinstitute.org/mammals/haploreg/haploreg.php), RegulomeDB (https://regulome.stanford.edu/regulome-search/), and FuncPred (https://manticore.niehs.nih.gov/snpinfo/snpfunc.html) were applied to identify potential functional SNPs in the human *RAD54L2* and *GTF2H1* genes.

Primer design and SNP genotyping were performed as shown in Suppl_Table 1. The genotyping primers were designed with the Agena MassARRAY Assay Design 3.0 software [17]. The Agena MassARRAY RS1000 system was used for genotyping, and the related data were managed using Agena Typer 4.0 software [17–19]. To ensure accuracy, about 5% of the samples were randomly re–genotyped, and the concordance of duplicated genotyping was 100%.

### Statistical analysis

Demographic characteristics between cases and controls were compared by Student’s t test and χ^2^ test. The Hardy-Weinberg equilibrium (HWE) was calculated by χ^2^ test [20]. Multiple genetic models were used to evaluate the association between gene polymorphisms and lung cancer risk. Odds ratios (ORs) and corresponding 95% confidence intervals (CIs) adjusting for age and gender were estimated using a logistic regression model through the PLINK software [21]. Further analyses based on age, gender, BMI, smoking, drinking, histology, lymph node metastasis, and clinical stages were performed to assess the impact of polymorphisms on lung cancer. Multifactor dimensionality reduction (MDR) (version 3.0.2) was applied to evaluate the impact of SNP–SNP interactions on the risk of lung cancer. The threshold of *p* was set at 0.05.

## Results

The information on patients with lung cancer and healthy participants was presented in Table 1. The mean ages of participants in the case and control groups were 59.80 ± 10.63 years and 59.80 ± 9.08 years, respectively. The case group includes 350 males and 156 females, and the control group consists of 353 males and 157 females. There were no significant differences in terms of age (*p* = 0.992) and gender (*p* = 0.987) between the two groups. Of the 506 patients, 269 had lymph node metastasis,103 had no metastasis, and 286 (56.50%) patients were in stage III–IV. And there were 174 (34.4%) cases of squamous cell carcinoma and 212 (41.9%) cases of adenocarcinoma.

**Table 1:** The information of the participants

Characteristics	Case	Control	*p*
Number	506	510	
Age (mean ± SD, years)	59.80 ± 10.63	59.80 ± 9.08	0.992
> 59	271 (53.6%)	275 (54.3%)	
≤ 59	235 (46.4%)	235 (46.4%)	
Gender			0.987
Male	350(69.2%)	353(69.8%)	
Female	156(30.8%)	157(31.2%)	
BMI (kg/m^2^)			
≤ 24	133 (26.3%)	138 (27.3%)	
> 24	81 (16.0%)	181 (35.8%)	
Missing	292 (57.7%)	191 (37.7%)	
Smoking			
Yes	242 (47.8%)	108 (27.3%)	
No	161 (31.8%)	180 (35.8%)	
Missing	292 (20.4%)	191 (43.5%)	
Drinking			
Yes	109 (21.5%)	103 (20.4%)	
No	267 (52.8%)	156 (30.8%)	
Missing	130 (25.7%)	251 (49.6%)	
Pathological type			
Lung squamous cell carcinoma	174 (34.4%)		
Lung adenocarcinoma	212 (41.9%)		
Missing	120 (23.7%)		
Lymph node metastasis			
Yes	269 (53.2%)		
No	103 (20.4%)		
Missing	134 (26.5%)		
Clinical stages			
I + II	93 (18.4)		
III + IV	286 (56.5)		
Missing	127 (25.1%)		

*p* values were calculated by χ^2^ test or the Student’s t test

Table 2 showed the basic information on nine SNPs in the *RAD54L2* and *GTF2H1* genes, including physical location, chromosome, minor allele frequency, and HWE. And all variants met the HWE. Associations between *RAD54L2* and *GTF2H1* polymorphisms and the risk of lung cancer were evaluated under different genetic models. In allele model (Table 2), no significant association of SNPs with the genetic susceptibility of lung cancer was found. Database analysis presented that the potential functions of these SNPs might be related to promoter /enhancer histone marks, transcription factor binding, DNAse, proteins binding, changed motifs changed, and selected expression quantitative trait loci (eQTL) hits.

**Table 2:** The information of nine gene polymorphisms on the *RAD54L2* and *GTF2H1* gene

Gene	SNP	Chromosome	Physical location	Alleles	MAF-Case	MAF-Control	HWE-*p*	OR (95%CI)	*p*	HaploReg v4.1	RegulomeDB	FuncPred
*RAD54L2*	rs11720298	3	51,547,493	A/G	0.25	0.28	0.229	0.86 (0.70–1.05)	0.127	Selected eQTL hits	TF binding + DNase peak	
	rs4687721	3	51,564,238	A/G	0.07	0.07	0.747	0.99 (0.71–1.39)	0.972	Promoter histone marks, Enhancer histone marks, Motifs changed, Selected eQTL hits	TF binding + any motif + DNase peak	
	rs4687592	3	51,621,840	C/T	0.34	0.33	0.159	1.07 (0.89–1.28)	0.490	Enhancer histone marks, DNAse, Proteins bound, Selected eQTL hits	TF binding + DNase peak	
	rs9864693	3	51,622,354	C/G	0.45	0.42	0.856	1.10 (0.92–1.31)	0.293	SiPhy cons, Promoter histone marks, Enhancer histone marks, Motifs changed, Selected eQTL hits	TF binding or DNase peak	
*GTF2H1*	rs4150530	11	18,322,147	G/T	0.10	0.12	0.831	0.84 (0.63–1.11)	0.219	Promoter histone marks, DNAse, Proteins bound, Motifs changed, Selected eQTL hits	TF binding + any motif + DNase Footprint + DNase peak	TFBS, Splicing
	rs3802967	11	18,322,517	A/G	0.46	0.48	0.479	0.92 (0.77–1.09)	0.323	SiPhy cons, Promoter histone marks, DNAse, Proteins bound, Motifs changed, Selected eQTL hits	TF binding + matched TF motif + matched DNase Footprint + DNase peak	TFBS
	rs4150606	11	18,342,022	A/C	0.13	0.14	0.591	0.91 (0.71–1.18)	0.485	Motifs changed, Selected eQTL hits	Motif hit	
	rs4150658	11	18,357,336	A/G	0.10	0.12	1.000	0.85 (0.64–1.12)	0.253	Motifs changed, Selected eQTL hits	TF binding or DNase peak	
	rs4150667	11	18,361,364	C/T	0.33	0.32	0.919	1.05 (0.87–1.27)	0.583	DNAse, Motifs changed, Selected eQTL hits	TF binding + DNase peak	

*SNP* Single nucleotide polymorphism, *MAF* Minor allele frequency, *HWE* Hardy-Weinberg equilibrium, *eQTL* expression Quantitative Trait Loci, *TF* Transcription factor, *TFBS* Transcription factor binding site

Haploreg (https://pubs.broadinstitute.org/mammals/haploreg/haploreg.php); RegulomeDB (https://regulome.stanford.edu/regulome-search/); FuncPred (https://manticore.niehs.nih.gov/snpinfo/snpfunc.html)

*p* values were calculated by χ^2^ test

In genotype model, subjects with *RAD54L2* rs9864693 GC heterozygote genotype might have an increased risk of lung cancer compared with individuals with GG wild-type genotype (crude analysis: OR = 1.33, 95%CI: 1.01–1.76, *p* = 0.046; adjusted analysis: OR = 1.33, 95%CI: 1.01–1.77, *p* = 0.045) (Table 3). No statistically significant association between other SNPs with lung cancer susceptibility was observed (*p* > 0.05, Suppl_Table 2).

**Table 3:** Risk analysis for*RAD54L2* rs9864693 and lung cancer in different genetic models by logistic regression analysis

SNP	Model	Genotype	control	case	crude analysis		adjusted analysis	
					OR (95% CI)	*p*-value	OR (95% CI)	*p*-value
*RAD54L2* rs9864693	Genotype	GG	171	143	1		1	
		GC	247	275	1.33 (1.01–1.76)	0.046*	1.33 (1.01–1.77)	0.045*
		CC	92	88	1.14 (0.79–1.65)	0.473	1.14 (0.79–1.65)	0.472
	Dominant	GG	171	143	1		1	
		GC-CC	339	363	1.28 (0.98–1.67)	0.069	1.28 (0.98–1.67)	0.069
	Recessive	GG-GC	418	418	1		1	
		CC	92	88	0.96 (0.69–1.32)	0.787	0.96 (0.69–1.32)	0.787
	Additive	-	-	-	1.10 (0.92–1.32)	0.282	1.10 (0.92–1.32)	0.282

*SNP* single nucleotide polymorphism, *OR* odds ratio, *95% CI* 95% confidence interval

*p* values were calculated by logistic regression analysis without/with adjustments for age and gender

*p < 0.05 respects the data is statistically significant

Stratified analysis was carried out based on age, gender, BMI, smoking, drinking, pathological type, lymph node metastasis, and clinical stages (Table 4). In subjects aged ≤ 59 years, *RAD54L2* rs11720298 was related to a reduced susceptibility to lung cancer, whereas several risk-increasing associations of *RAD54L2* rs4687592 (OR = 0.69, *p* = 0.012), *RAD54L2* rs9864693 (OR = 1.64, *p* = 0.012) and *GTF2H1* rs4150667 (OR = 1.32, *p* = 0.048) with lung cancer were found. Among subjects with BMI ≤ 24 kg/m^2^, *GTF2H1* rs4150667 (OR = 0.18, *p* = 0.008) contributed to a lower risk of developing lung cancer. Among non-smokers and drinkers, the protective effects of *RAD54L2* rs11720298 (OR = 0.62, *p* = 0.011; and OR = 0.17, *p* = 0.008) on the occurrence of lung cancer were observed. Besides, an increased risk of lung cancer was observed for *RAD54L2* rs4687592 in drinkers (OR = 2.54, *p* = 0.034). Stratified by pathological type, *GTF2H1* rs3802967 was associated with a reduced risk of lung squamous cell carcinoma (OR = 0.68, *p* = 0.045). However, no significant relationships of selected polymorphisms with lung cancer risk in the stratified analysis by gender, lymph node metastasis, and clinical stages were detected (Suppl_Table 3 and Suppl_Table 4).

**Table 4:** Stratified analysis for the associations between *RAD54L2* and *GTF2H1* polymorphisms and the risk of lung cancer

SNP	Model	Genotype	Number		adjusted by age and sex		Number		adjusted by age and sex	
			Case	Control	OR (95%CI)	*p*	Case	Control	OR (95%CI)	*p*
**Age ≤ 59 years**							**Age > 59 years**			
*RAD54L2* rs11720298	Genotype	AA	143	115	1		143	153	1	
		GA	77	98	0.61 (0.41–0.90)	0.013*	108	87	1.16 (0.81–1.67)	0.408
		GG	15	21	0.57 (0.28–1.16)	0.120	20	25	0.8 (0.42–1.51)	0.489
	dominant	AA/GG + GA	143/92	115/119	0.60 (0.42–0.87)	0.007*	143/128	153/122	1.09 (0.77–1.53)	0.625
	recessive	GA + AA/GG	220/15	213/21	0.70 (0.35–1.39)	0.306	251/20	250/25	0.75 (0.40–1.39)	0.362
	log-additive				0.69 (0.51–0.92)	0.012*			1.00 (0.77–1.30)	0.991
*RAD54L2* rs4687592	Genotype	CC	106	129	1		120	110	1	
		TC	96	82	1.41 (0.95–2.10)	0.085	120	128	0.85 (0.59–1.22)	0.377
		TT	33	24	1.74 (0.96–3.13)	0.066	31	37	0.78 (0.45–1.34)	0.366
	dominant	CC/TT + TC	106/129	129/106	1.49 (1.03–2.14)	0.034*	120/151	110/165	0.83 (0.59–1.17)	0.297
	recessive	TC + CC/TT	202/33	211/24	1.50 (0.85–2.63)	0.162	240/31	238/37	0.85 (0.51–1.41)	0.522
	log-additive				1.35 (1.03–1.76)	0.028*			0.87 (0.68–1.12)	0.284
*RAD54L2* rs9864693	Genotype	CC	70	97	1		73	74	1	
		GC	119	101	1.60 (1.06–2.40)	0.025*	156	146	1.09 (0.73–1.61)	0.688
		GG	46	37	1.75 (1.03–2.99)	0.039*	42	55	0.80 (0.47–1.34)	0.391
	dominant	CC/GG + GC	70/165	97/138	1.64 (1.12–2.40)	0.012*	73/198	74/201	1.01 (0.69–1.47)	0.972
	recessive	GC + CC/GG	189/46	198/37	1.35 (0.83–2.17)	0.226	229/42	220/55	0.75 (0.48–1.18)	0.215
	log-additive				1.37 (1.05–1.77)	0.019*			0.91 (0.71–1.18)	0.488
*GTF2H1* rs4150667	Genotype	CC	98	118	1		127	120	1	
		TC	109	97	1.38 (0.94–2.03)	0.100	122	125	0.92 (0.64–1.31)	0.626
		TT	28	20	1.67 (0.88–3.15)	0.115	22	30	0.67 (0.36–1.23)	0.194
	dominant	CC/TT + TC	98/137	118/117	1.43 (0.99–2.07)	0.055	127/144	120/155	0.87 (0.62–1.22)	0.411
	recessive	TC + CC/TT	207/28	215/20	1.42 (0.78–2.61)	0.254	249/22	245/30	0.70 (0.39–1.25)	0.227
	log-additive				1.32 (1.00-1.75)	0.048*			0.85 (0.66–1.11)	0.238
**BMI ≤ 24 kg/m**^**2**^							**BMI > 24 kg/m**^**2**^			
*GTF2H1* rs4150667	Genotype	CC	68	65	1		33	93	1	
		TC	62	58	1.02 (0.62–1.67)	0.955	37	76	1.36 (0.77–2.40)	0.286
		TT	3	15	0.18 (0.05–0.65)	0.009*	11	12	2.44 (0.97–6.18)	0.059
	dominant	CC/TT + TC	68/65	65/73	0.84 (0.52–1.36)	0.474	33/48	93/88	1.51 (0.88–2.58)	0.135
	recessive	TC + CC/TT	130/3	123/15	0.18 (0.05–0.63)	0.008*	70/11	169/12	2.09 (0.87–5.06)	0.100
	log-additive				0.70 (0.47–1.04)	0.077			1.49 (0.99–2.24)	0.059
**Non-smokers**							**Smokers**			
*RAD54L2* rs11720298	Genotype	AA	92	88	1		138	56	1	
		GA	62	75	0.72 (0.45–1.15)	0.167	86	41	0.84 (0.52–1.37)	0.485
		GG	7	16	0.28 (0.11–0.75)	0.011*	18	11	0.69 (0.30–1.55)	0.367
	dominant	AA/GG + GA	92/69	88/91	0.63 (0.40–0.99)	0.047*	138/104	56/52	0.81 (0.51–1.28)	0.362
	recessive	GA + AA/GG	154/7	163/16	0.33 (0.13–0.85)	0.021*	224/18	97/11	0.74 (0.33–1.63)	0.448
	log-additive				0.62 (0.43–0.89)	0.011*			0.83 (0.59–1.18)	0.305
**Drinkers**							**Non-drinkers**			
*RAD54L2* rs11720298	Genotype	AA	66	49	1		251	75	1	
		GA	40	41	0.70 (0.39–1.24)	0.217	95	66	0.72 (0.47–1.09)	0.118
		GG	3	13	0.17 (0.05–0.63)	0.008*	21	14	0.76 (0.36–1.57)	0.455
	dominant	AA/GG + GA	66/43	49/54	0.57 (0.33–0.99)	0.045	151/116	75/80	0.72 (0.48–1.08)	0.110
	recessive	GA + AA/GG	106/3	90/13	0.20 (0.05–0.71)	0.013	246/21	141/14	0.87 (0.43–1.78)	0.708
	log-additive				0.54 (0.35–0.85)	0.008*			0.80 (0.59–1.09)	0.162
*RAD54L2* rs4687592	Genotype	CC	38	47	1		124	77	1	
		TC	50	45	1.37 (0.76–2.48)	0.295	114	66	1.08 (0.71–1.65)	0.706
		TT	21	11	2.54 (1.07-6.00)	0.034*	29	13	1.43 (0.70–2.92)	0.329
	dominant	CC/TT + TC	38/71	47/56	1.60 (0.91–2.79)	0.101	124/143	77/79	1.14 (0.77–1.70)	0.518
	recessive	TC + CC/TT	88/21	92/11	2.15 (0.97–4.79)	0.061	238/29	143/13	1.38 (0.69–2.74)	0.365
	log-additive				1.53 (1.03–2.28)	0.035*			1.15 (0.85–1.56)	0.366
**Lung squamous cell carcinoma**							**Lung adenocarcinoma**			
*GTF2H1* rs3802967	Genotype	CC	59	133	1		65	133	1	
		TC	79	263	0.67 (0.45-1.00)	0.051	96	263	0.74 (0.51–1.09)	0.128
		TT	36	114	0.70 (0.43–1.15)	0.158	51	114	0.94 (0.60–1.47)	0.780
	dominant	CC/TT + TC	59/115	133/377	0.68 (0.46–0.99)	0.045*	65/147	133/377	0.80 (0.56–1.14)	0.224
	recessive	TC + CC/TT	138/36	396/114	0.90 (0.59–1.38)	0.629	161/51	396/114	1.13 (0.77–1.66)	0.526
	log-additive				0.82 (0.64–1.05)	0.118			0.96 (0.76–1.20)	0.694

*SNP* single nucleotide polymorphism, *OR* odds ratio, *95% CI* 95% confidence interval

*p* values were calculated by logistic regression analysis with adjustments for age and gender

*p < 0.05 respects the data is statistically significant

FPRP analysis was performed to verify positive findings, as shown in Table 5. At a prior probability level of 0.25, a significant association of *RAD54L2* rs9864693 remained noteworthy overall (FPRP = 0.160 and statistical power = 0.795). In subjects aged ≤ 59 years, correlations of rs11720298, rs4687592 and rs9864693 in *RAD54L2* with the susceptibility to lung cancer were also positive at a prior probability level of 0.1. Moreover, an association of *RAD54L2* rs11720298 with the risk of lung cancer in non-smokers and drinkers was significant at a prior probability level of 0.1.

**Table 5:** False-positive report probability values for the associations between *RAD54L2* and *GTF2H1* polymorphisms and the risk of lung cancer

SNP	Model	adjusted by age and sex		Statistical power	Prior probability				
		OR (95%CI)	*p*		0.25	0.1	0.01	0.001	0.0001
**Overall**									
*RAD54L2* rs9864693	Genotype	1.33 (1.01–1.77)	0.045	0.795	0.160^a^	0.364	0.863	0.984	0.998
**Age ≤ 59 years**									
*RAD54L2* rs11720298	Genotype	0.61 (0.41–0.90)	0.013	0.842	0.043^a^	0.120^a^	0.600	0.938	0.993
	dominant	0.60 (0.42–0.87)	0.007	0.832	0.025^a^	0.071^a^	0.456	0.894	0.988
	log-additive	0.69 (0.51–0.92)	0.012	0.986	0.034^a^	0.095^a^	0.535	0.921	0.991
*RAD54L2* rs4687592	dominant	1.49 (1.03–2.14)	0.034	0.944	0.089^a^	0.227	0.764	0.970	0.997
	log-additive	1.35 (1.03–1.76)	0.028	0.998	0.074^a^	0.193^a^	0.725	0.964	0.996
*RAD54L2* rs9864693	Genotype	1.60 (1.06–2.40)	0.025	0.860	0.075^a^	0.195^a^	0.727	0.964	0.996
		1.75 (1.03–2.99)	0.039	0.687	0.150^a^	0.347	0.854	0.983	0.998
	dominant	1.64 (1.12–2.40)	0.012	0.846	0.037^a^	0.104^a^	0.560	0.928	0.992
	log-additive	1.37 (1.05–1.77)	0.019	0.756	0.060^a^	0.160a	0.677	0.955	0.995
*GTF2H1* rs4150667	log-additive	1.32 (1.00–1.75)	0.048	0.813	0.165^a^	0.373	0.867	0.985	0.998
**BMI ≤ 24 kg/m**^**2**^									
*GTF2H1* rs4150667	Genotype	0.18 (0.05–0.65)	0.009	0.059	0.309	0.573	0.937	0.993	0.999
	recessive	0.18 (0.05–0.63)	0.008	0.055	0.285	0.544	0.929	0.993	0.999
**Non-smokers**									
*RAD54L2* rs11720298	Genotype	0.28 (0.11–0.75)	0.011	0.124	0.215	0.451	0.900	0.989	0.999
	dominant	0.63 (0.40–0.99)	0.047	0.842	0.138^a^	0.325	0.841	0.982	0.998
	recessive	0.33 (0.13–0.85)	0.021	0.195	0.250	0.500	0.917	0.991	0.999
	log-additive	0.62 (0.43–0.89)	0.011	0.878	0.032^a^	0.089^a^	0.518	0.916	0.991
**Drinkers**									
*RAD54L2* rs11720298	Genotype	0.17 (0.05–0.63)	0.008	0.053	0.311	0.575	0.937	0.993	0.999
	log-additive	0.54 (0.35–0.85)	0.008	0.630	0.036^a^	0.100^a^	0.549	0.925	0.992
*RAD54L2* rs4687592	Genotype	2.54 (1.07–6.00)	0.034	0.293	0.256	0.508	0.919	0.991	0.999
	log-additive	1.53 (1.03–2.28)	0.035	0.906	0.108^a^	0.267	0.800	0.976	0.998
**Lung squamous cell carcinoma**									
*GTF2H1* rs3802967	dominant	0.68 (0.46–0.99)	0.045	0.946	0.123^a^	0.296	0.822	0.979	0.998

*SNP* single nucleotide polymorphism, *OR* odds ratio, *95% CI* 95% confidence interval

*p* values were calculated by logistic regression analysis with adjustments for age

Statistical power was calculated using the number of observations in the subgroup and the OR and *p* values in this table

^a^The level of false-positive report probability threshold was set at 0.2, and noteworthy findings are presented

**Fig. 1:**
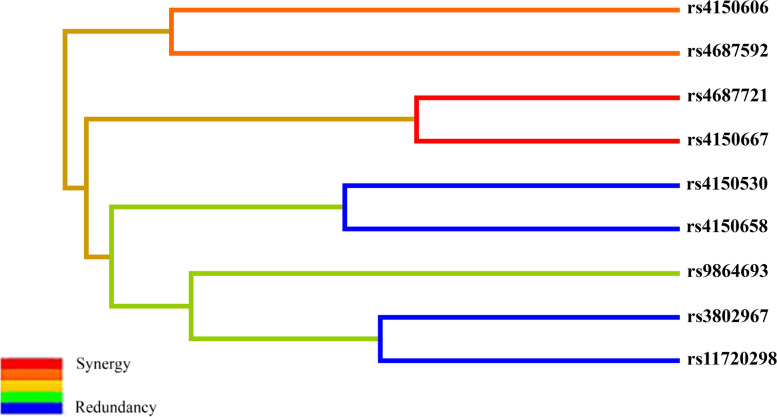
Dendrogram for the interactions among SNPs in *GTF2H1* and *RAD54L2* on the risk of lung cancer.  Red and orange lines indicate synergistic interaction, blue and green color indicated redundant interactions. Short connections among nodes represent stronger interactions

MDR was applied to analyze the interactions of these SNPs. The results of MDR model analysis for SNP-SNP interactions were displayed in Table 6; Fig. 1. The best multi-loci model was the eight-locus model, namely, a combination of rs3802967, rs4687721, rs9864693, rs11720298, rs4150606, rs4150658, rs4150667, and rs4687592, with a highest testing accuracy (0.5286) and a perfect cross-validation consistency (10/10). As shown in Fig. 1, the dendrogram plot demonstrated the interactions among these eight SNPs and recapitulated the main and/or interaction effect on each pairwise combination of attributes. Red and orange line indicated synergistic interaction, blue and green color indicated redundant interactions. The result suggested that rs4687721 and rs4150667 had a synergistic interaction sharing the positive information gain with lung cancer.

**Table 6:** SNP–SNP interaction models of candidate SNPs analyzed by the MDR method

Model	Training Bal. Acc.	Testing Bal. Acc.	CVC	OR (95% CI)	*p*
rs9864693	0.5315	0.499	8/10	1.28 (1.00-1.63)	0.0517
rs3802967,rs9864693	0.5428	0.4824	4/10	1.42 (1.10–1.85)	0.0078*
rs3802967,rs9864693,rs4150658	0.5628	0.5207	8/10	1.67 (1.29–2.14)	< 0.0001*
rs3802967,rs4687721,rs9864693,rs4150658	0.5815	0.4855	3/10	1.99 (1.53–2.58)	< 0.0001*
rs3802967,rs9864693,rs11720298,rs4150658,rs4687592	0.6098	0.5158	5/10	2.47 (1.89–3.23)	< 0.0001*
rs3802967,rs9864693,rs11720298,rs4150658,rs4150667,rs4687592	0.6344	0.5276	8/10	2.92 (2.62–3.76)	< 0.0001*
rs3802967,rs9864693,rs11720298,rs4150606,rs4150658,rs4150667,rs4687592	0.644	0.5217	9/10	3.13 (2.42–4.04)	< 0.0001*
rs3802967,rs4687721,rs9864693,rs11720298,rs4150606,rs4150658,rs4150667,rs4687592	0.6507	0.5286	10/10	3.34 (2.58–4.32)	< 0.0001*
rs3802967,rs4687721,rs9864693,rs11720298,rs4150530,rs4150606,rs4150658,rs4150667,rs4687592	0.6523	0.5306	10/10	3.40 (2.62–4.40)	< 0.0001*

*MDR* multifactor dimensionality reduction, *Bal. Acc.* balanced accuracy, *CVC* cross–validation consistency, *OR* odds ratio, *CI* confidence interval

*p* values were calculated using χ^2^ tests

Bold indicate that *p* < 0.05 indicates statistical significance

## Discussion

In our research, we found that *RAD54L2* rs9864693 was related to an increased risk of lung cancer in the Chinese Han population. Especially, among subjects aged ≤ 59 years, *RAD54L2* rs11720298 was related to a reduced susceptibility to lung cancer, while the risk-increasing associations were found for *RAD54L2* rs4687592, *RAD54L2* rs9864693 and *GTF2H1* rs4150667. Among subjects with BMI ≤ 24 kg/m^2^, *GTF2H1* rs4150667 had the lower risk of developing lung cancer. In non-smokers and drinkers, the protective risk effect of *RAD54L2* rs11720298 on the occurrence of lung cancer was observed, respectively. Besides, the increased risk of lung cancer was observed for *RAD54L2* rs4687592 in drinkers. *GTF2H1* rs3802967 was associated with the reduced risk of lung squamous cell carcinoma. MDR result suggested that rs4687721 and rs4150667 had synergistic interaction sharing the positive information gain with respect to lung cancer. Our findings provide data for constructing a genetic panel to predict lung cancer risk in China.

*RAD54L2* (ARIP4), located on human chromosome 3p21.2, is initially identified as an ATPase of Rad54/ATRX subfamily of SNF2-like proteins, which contains chromatin remodeling activity, interacts with AR and regulates androgen-mediated transactivation [22]. SNF2-like proteins are thought to modify the structure of chromatin in a non-covalent manner through rearrangement of nucleosomes. Only *RAD54L2* has been found to affect gastrointestinal stromal tumors, and the expression of *RAD54L2* has a shorter overall survival time [23]. *Rad54* interacts with *Rad51*, which functions during DNA repair. It has been proved that *Rad51* G135C allele is associated with a higher survival time and a better prognosis in lung cancer patients treated with platinum-paclitaxel / gemcitabine Wrst line chemotherapy [16]. RAD54B protein expression is significantly higher in lung adenocarcinoma tissues than that in healthy lung tissues, and inhibition of *RAD54B* expression in A549 cells can significantly reduce cell proliferation and increase apoptotic rate [24]. However, the effect of *RAD54L2* on lung cancer is not clear. In our research, we found that *RAD54L2* rs9864693 was associated with an increased risk of lung cancer, especially in subjects aged ≤ 59 years. Besides, *RAD54L2* rs11720298 was related to a reduced susceptibility to lung cancer in subjects aged ≤ 59 years, non-smokers and drinkers, while a risk-increasing association was found between rs4687592 and lung cancer risk in subjects aged ≤ 59 years and drinkers. Here, we first displayed the genetic association of *RAD54L2* polymorphisms with the susceptibility to lung cancer in the Chinese Han population. However, the role of *RAD54L2* gene in the occurrence and development of lung cancer needs to be further clarified.

*GTF2H1* interacts with the C- and N-terminus of XPC protein, participates in the recruitment of other protein subunits in TFIIH, and initiates the NER repair process. The study found that the expression of *GTF2H1* was down-regulated in lung cancer tissues [25]. Rs3802967 is located in the 5’-UTR region of *GTF2H1* gene, and the luciferase activity experiments displayed that rs3802967 T allele was related to the enhanced expression of *GTF2H1* in lung cancer cells [14]. A previous study has showed that variants of *GTF2H1* rs3802967 and rs4150667 are also significantly associated with the risk of lung cancer in the southern Han Chinese population [14]. In our study, it was found that *GTF2H1* rs3802967 CT-TT reduced the risk of lung squamous cell carcinoma. Moreover, a risk-increasing association was found between *GTF2H1* rs4150667 and lung cancer risk in subjects aged ≤ 59 years, while *GTF2H1* rs4150667 was associated with a reduced risk of developing lung cancer in subjects with BMI ≤ 24 kg/m^2^. However, such inconsistency might result from different behavioral habits or sample sizes. Besides, rs4150606 on *GTF2H1* increased the risk of lung cancer [14]. However, no correlation between the variants of *GTF2H1* rs4150606 and the risk of lung cancer was found. This may be due to the false negative results of our small sample size, and whether the association of SNPs with the risk of lung cancer needs to be further confirmed.

Several limitations in this study should be considered. First, the subjects were enrolled from the same hospital; therefore, there is selection bias. Second, after stratification, the sample size of each subgroup is relatively small, thus, a well-designed large sample is needed to further confirm our findings. Third, the mechanism of selected polymorphisms on the occurrence and development of lung cancer is still unclear, and further research is needed. Fourth, due to the absence of information, including BMI, smoking, and drinking, only age- and sex- adjustments were performed in this study. In the follow-up studies, we will improve relevant information and adjust risk factors to further analyze the correlation of *GTF2H1* and *RAD54L2* with the risk of lung cancer.

## Conclusion

To sum up, our study first revealed that *RAD54L2* rs9864693 was associated with an increased risk of lung cancer in the Chinese Han population. Our finding will provide evidence that age, BMI, smoking, and drinking might be associated with the effects of *RAD54L2* and *GTF2H1* variants on lung cancer susceptibility. This study may increase the understanding the effects of of *RAD54L2* and *GTF2H1* polymorphisms on the occurrence of lung cancer.

## Supplementary Information

**Additional file 1: Suppl_Table 1**. The detail of PCR primers and UEP sequence for candidate variants. **Suppl_Table 2.** Risk analysis for RAD54L2 and GTF2H1 polymorphisms with the susceptibility to lung cancer in different genetic models by logistic regression analysis. **Suppl_Table 3.** Stratified analysis for the associations between RAD54L2 and GTF2H1 polymorphisms and the risk of lung cancer.  **Suppl_Table 4.** The associations between RAD54L2 and GTF2H1 polymorphisms and the stage and metastasis of lung cancer. 

## Data Availability

The data that support the findings in this study are available on request from the first author and correspondent author. The data are not publicly available as they contain information that could compromise research participant privacy or consent.
