# Supplementary material for: Impact of GTF2H1 and RAD54L2 polymorphisms on the risk of lung cancer in the Chinese Han population
Source: BMC Cancer. 2022 Nov 16;22:1181. doi: 10.1186/s12885-022-10303-1 (PMC9670636; doi:10.1186/s12885-022-10303-1)
Supplement: Supplementary file 1 — Additional file 1: Suppl_Table 1. The detail of PCR primers and UEP sequence for candidate variants. Suppl_Table 2. Risk analysis for RAD54L2 and GTF2H1 polymorphisms with the susceptibility to lung cancer in different genetic models by logistic regression analysis. Suppl_Table 3. Stratified analysis for the associations between RAD54L2 and GTF2H1 polymorphisms and the risk of lung cancer. Suppl_Table 4. The associations between RAD54L2 and GTF2H1 polymorphisms and the stage and metastasis of lung cancer. [file 12885_2022_10303_MOESM1_ESM.docx]

Suppl_Table 1. The detail of PCR primers and UEP sequence for candidate variants

| Gene | SNPs | First Primer(5'-3') | Second Primer (5'-3') | UEP_DIR | | | UEP SEQ (5'-3') |
| --- | --- | --- | --- | --- | --- | --- | --- |
| *RAD54L2* | rs11720298 | ACGTTGGATGGGCACATTGGAGGTACTAAC | ACGTTGGATGGCAAACATTTTTCATTTCCGC | | F | ggGCTTATGGTTTTATAGTACCATCA | |
| *RAD54L2* | rs4687721 | ACGTTGGATGTGAAGCAGTATGTCTCTGCC | ACGTTGGATGCTAATGGAGGACTCCAAATC | | F | ACACAGGAAAACAGGGA | |
| *RAD54L2* | rs4687592 | ACGTTGGATGAAGTTGAAGTGTGCCAACCC | ACGTTGGATGCACCAATACTGCAACTTGGC | | F | caaaCCAGCCTCAAGTGTAGT | |
| *RAD54L2* | rs9864693 | ACGTTGGATGTCACACACGCAGTACATTCC | ACGTTGGATGTACCACCGGTCTGTAATAGG | | F | CCTAGCCTCTGCTTTCTTT | |
| *GTF2H1* | rs4150530 | ACGTTGGATGATGACGTCAGCAAGGAAGGG | ACGTTGGATGGATCTTGTCTCCCGGCTTAG | | R | tGGGAGACTGAAGTTCACGTTC | |
| *GTF2H1* | rs3802967 | ACGTTGGATGACGTGACCGGAAGTAGCAAG | ACGTTGGATGACTAGCGGAAGGTGTCATGG | | R | ttgtCGGGCCCTGGGCACGTGACTCA | |
| *GTF2H1* | rs4150606 | ACGTTGGATGCTGAGGTAGGTAGGTCATAC | ACGTTGGATGGCCAAACCTTAAGCACTCTC | | R | TTGCTGAAACATTGGGGTTG | |
| *GTF2H1* | rs4150658 | ACGTTGGATGTACCCTTGATAACAGAGTGG | ACGTTGGATGGTTGATGTTGATACAGTGGG | | R | TTAATTTTCAAGGAAGTGAATAAA | |
| *GTF2H1* | rs4150667 | ACGTTGGATGCTCCAGAGGTCATGACTAAC | ACGTTGGATGACCCGGCCAGATTGTAAAAC | | F | AACCATCTCCATGCTTTCC | |

SNP, Single nucleotide polymorphism; UEP, Unextended mini sequencing primer; DIR, direction; SEQ, sequence.

Suppl_Table 2. Risk analysis for *RAD54L2* and *GTF2H1* polymorphisms with the susceptibility to lung cancer in different genetic models by logistic regression analysis

| SNP | Model | Genotype | Number | | Crude analysis | | adjusted by age and sex | |
| --- | --- | --- | --- | --- | --- | --- | --- | --- |
|  |  |  | Case | Control | OR (95%CI) | p | OR (95%CI) | p |
| *RAD54L2*  rs11720298 | Genotype | AA | 286 | 268 | 1 |  | 1 |  |
|  |  | GA | 185 | 195 | 0.89 (0.68-1.15) | 0.377 | 0.89 (0.68-1.15) | 0.373 |
|  |  | GG | 35 | 46 | 0.71 (0.45-1.14) | 0.158 | 0.71 (0.44-1.14) | 0.157 |
|  | dominant | AA/GG+GA | 286/220 | 268/241 | 0.86 (0.67-1.10) | 0.216 | 0.85 (0.67-1.10) | 0.213 |
|  | recessive | GA+AA/GG | 471/35 | 463/46 | 0.75 (0.47-1.18) | 0.214 | 0.75 (0.47-1.18) | 0.213 |
|  | log-additive |  |  |  | 0.86 (0.71-1.05) | 0.136 | 0.86 (0.71-1.05) | 0.134 |
| *RAD54L2*  rs4687721 | Genotype | AA | 435 | 438 | 1 |  | 1 |  |
|  |  | GA | 68 | 69 | 0.99 (0.69-1.42) | 0.967 | 0.99 (0.69-1.42) | 0.966 |
|  |  | GG | 3 | 3 | 1.01 (0.20-5.02) | 0.993 | 1.01 (0.20-5.04) | 0.993 |
|  | dominant | AA/GG+GA | 435/71 | 438/72 | 0.99 (0.70-1.41) | 0.969 | 0.99 (0.70-1.41) | 0.968 |
|  | recessive | GA+AA/GG | 503/3 | 507/3 | 1.01 (0.20-5.02) | 0.992 | 1.01 (0.20-5.04) | 0.992 |
|  | log-additive |  |  |  | 0.99 (0.71-1.39) | 0.972 | 0.99 (0.71-1.39) | 0.972 |
| *RAD54L2*  rs4687592 | Genotype | CC | 226 | 239 | 1 |  | 1 |  |
|  |  | TC | 216 | 210 | 1.09 (0.84-1.42) | 0.531 | 1.09 (0.84-1.42) | 0.529 |
|  |  | TT | 64 | 61 | 1.11 (0.75-1.65) | 0.606 | 1.11 (0.75-1.65) | 0.604 |
|  | dominant | CC/TT+TC | 226/280 | 239/271 | 1.09 (0.85-1.40) | 0.482 | 1.09 (0.85-1.40) | 0.48 |
|  | recessive | TC+CC/TT | 442/64 | 449/61 | 1.07 (0.73-1.55) | 0.739 | 1.07 (0.73-1.55) | 0.738 |
|  | log-additive |  |  |  | 1.06 (0.89-1.27) | 0.502 | 1.06 (0.89-1.27) | 0.5 |
| *GTF2H1*  rs4150530 | Genotype | GG | 409 | 399 | 1 |  | 1 |  |
|  |  | TG | 94 | 104 | 0.88 (0.65-1.20) | 0.428 | 0.88 (0.65-1.20) | 0.426 |
|  |  | TT | 3 | 7 | 0.42 (0.11-1.63) | 0.209 | 0.42 (0.11-1.63) | 0.207 |
|  | dominant | GG/TT+TG | 409/97 | 399/111 | 0.85 (0.63-1.16) | 0.306 | 0.85 (0.63-1.16) | 0.305 |
|  | recessive | TG+GG/TT | 503/3 | 503/7 | 0.43 (0.11-1.67) | 0.221 | 0.43 (0.11-1.67) | 0.221 |
|  | log-additive |  |  |  | 0.84 (0.63-1.11) | 0.216 | 0.83 (0.63-1.11) | 0.215 |
| *GTF2H1*  rs3802967 | Genotype | CC | 153 | 133 | 1 |  | 1 |  |
|  |  | TC | 241 | 263 | 0.80 (0.60-1.07) | 0.125 | 0.80 (0.60-1.07) | 0.125 |
|  |  | TT | 112 | 114 | 0.85 (0.60-1.21) | 0.376 | 0.85 (0.60-1.21) | 0.376 |
|  | dominant | CC/TT+TC | 153/353 | 133/377 | 0.81 (0.62-1.07) | 0.141 | 0.81 (0.62-1.07) | 0.141 |
|  | recessive | TC+CC/TT | 394/112 | 396/114 | 0.99 (0.73-1.33) | 0.933 | 0.99 (0.73-1.33) | 0.934 |
|  | log-additive |  |  |  | 0.92 (0.77-1.09) | 0.324 | 0.92 (0.77-1.09) | 0.324 |
| *GTF2H1*  rs4150606 | Genotype | AA | 379 | 375 | 1 |  | 1 |  |
|  |  | CA | 119 | 123 | 0.96 (0.72-1.28) | 0.768 | 0.96 (0.72-1.28) | 0.766 |
|  |  | CC | 8 | 12 | 0.66 (0.27-1.63) | 0.368 | 0.66 (0.27-1.63) | 0.367 |
|  | dominant | AA/CC+CA | 379/127 | 375/135 | 0.93 (0.70-1.23) | 0.617 | 0.93 (0.70-1.23) | 0.617 |
|  | recessive | CA+AA/CC | 498/8 | 498/12 | 0.67 (0.27-1.65) | 0.379 | 0.67 (0.27-1.65) | 0.379 |
|  | log-additive |  |  |  | 0.91 (0.71-1.18) | 0.486 | 0.91 (0.71-1.18) | 0.485 |
| *GTF2H1*  rs4150658 | Genotype | AA | 407 | 397 | 1 |  | 1 |  |
|  |  | GA | 95 | 106 | 0.87 (0.64-1.19) | 0.395 | 0.87 (0.64-1.19) | 0.393 |
|  |  | GG | 4 | 7 | 0.56 (0.16-1.92) | 0.354 | 0.56 (0.16-1.92) | 0.352 |
|  | dominant | AA/GG+GA | 407/99 | 397/113 | 0.85 (0.63-1.16) | 0.31 | 0.85 (0.63-1.16) | 0.309 |
|  | recessive | GA+AA/GG | 502/4 | 503/7 | 0.57 (0.17-1.97) | 0.376 | 0.57 (0.17-1.97) | 0.376 |
|  | log-additive |  |  |  | 0.85 (0.64-1.12) | 0.25 | 0.85 (0.64-1.12) | 0.249 |
| *GTF2H1*  rs4150667 | Genotype | CC | 225 | 238 | 1 |  | 1 |  |
|  |  | TC | 231 | 222 | 1.10 (0.85-1.43) | 0.468 | 1.10 (0.85-1.43) | 0.468 |
|  |  | TT | 50 | 50 | 1.06 (0.69-1.63) | 0.799 | 1.06 (0.69-1.63) | 0.799 |
|  | dominant | CC/TT+TC | 225/281 | 238/272 | 1.09 (0.85-1.40) | 0.481 | 1.09 (0.85-1.40) | 0.482 |
|  | recessive | TC+CC/TT | 456/50 | 460/50 | 1.01 (0.67-1.52) | 0.967 | 1.01 (0.67-1.53) | 0.967 |
|  | log-additive |  |  |  | 1.06 (0.87-1.27) | 0.578 | 1.06 (0.87-1.27) | 0.578 |

SNP, single nucleotide polymorphism; OR, odds ratio; 95% CI, 95% confidence interval.

*p* values were calculated by logistic regression analysis without/with adjustments for age and gender.

Suppl_Table 3. Stratified analysis for the associations between *RAD54L2* and *GTF2H1* polymorphisms and the risk of lung cancer

| SNP | Model | Genotype | Number | | adjusted by age and sex | | Number | | adjusted by age and sex | |
| --- | --- | --- | --- | --- | --- | --- | --- | --- | --- | --- |
|  |  |  | Case | Control | OR (95%CI) | p | Case | Control | OR (95%CI) | p |
|  |  |  | **Age > 59 years** | | | | **Age ≤ 59 years** | | | |
| rs4687721 | Genotype | AA | 234 | 232 | 1 |  | 201 | 206 | 1 |  |
|  |  | GA | 37 | 42 | 0.90 (0.56-1.45) | 0.663 | 31 | 27 | 1.16 (0.67-2.02) | 0.598 |
|  |  | GG | 0 | 1 | / | / | 3 | 2 | 1.39 (0.23-8.50) | 0.719 |
|  | dominant | AA/GG+GA | 234/37 | 232/43 | 0.88 (0.54-1.41) | 0.589 | 201/34 | 206/29 | 1.18 (0.69-2.01) | 0.549 |
|  | recessive | GA+AA/GG | 271/0 | 274/1 | / | / | 232/3 | 233/2 | 1.37 (0.22-8.32) | 0.734 |
|  | log-additive |  |  |  | 0.86 (0.54-1.37) | 0.515 |  |  | 1.17 (0.72-1.89) | 0.530 |
| rs4150530 | Genotype | GG | 217 | 210 | 1 |  | 192 | 189 | 1 |  |
|  |  | TG | 52 | 62 | 0.79 (0.52-1.20) | 0.276 | 42 | 42 | 0.98 (0.61-1.57) | 0.919 |
|  |  | TT | 2 | 3 | 0.62 (0.10-3.83) | 0.611 | 1 | 4 | 0.23 (0.03-2.11) | 0.194 |
|  | dominant | GG/TT+TG | 217/54 | 210/65 | 0.78 (0.52-1.18) | 0.248 | 192/43 | 189/46 | 0.91 (0.57-1.45) | 0.693 |
|  | recessive | TG+GG/TT | 269/2 | 272/3 | 0.66 (0.11-4.01) | 0.649 | 234/1 | 231/4 | 0.23 (0.03-2.11) | 0.195 |
|  | log-additive |  |  |  | 0.79 (0.54-1.17) | 0.237 |  |  | 0.86 (0.56-1.32) | 0.482 |
| rs3802967 | Genotype | CC | 84 | 80 | 1 |  | 47 | 55 | 1 |  |
|  |  | TC | 122 | 126 | 0.87 (0.58-1.28) | 0.472 | 119 | 127 | 1.14 (0.71-1.81) | 0.594 |
|  |  | TT | 65 | 59 | 1.07 (0.67-1.70) | 0.794 | 69 | 53 | 1.55 (0.91-2.64) | 0.107 |
|  | dominant | CC/TT+TC | 84/187 | 80/195 | 0.93 (0.64-1.34) | 0.680 | 47/188 | 55/180 | 1.26 (0.81-1.96) | 0.309 |
|  | recessive | TC+CC/TT | 206/65 | 216/59 | 1.16 (0.78-1.74) | 0.463 | 166/69 | 182/53 | 1.42 (0.93-2.15) | 0.102 |
|  | log-additive |  |  |  | 1.02 (0.81-1.29) | 0.870 |  |  | 1.25 (0.96-1.63) | 0.099 |
| rs4150606 | Genotype | AA | 201 | 201 | 1 |  | 178 | 174 | 1 |  |
|  |  | CA | 65 | 67 | 0.92 (0.62-1.38) | 0.697 | 54 | 56 | 0.94 (0.61-1.44) | 0.767 |
|  |  | CC | 5 | 7 | 0.74 (0.23-2.38) | 0.608 | 3 | 5 | 0.53 (0.12-2.30) | 0.399 |
|  | dominant | AA/CC+CA | 201/70 | 201/74 | 0.91 (0.62-1.33) | 0.617 | 178/57 | 174/61 | 0.90 (0.59-1.38) | 0.636 |
|  | recessive | CA+AA/CC | 266/5 | 268/7 | 0.75 (0.23-2.41) | 0.629 | 232/3 | 230/5 | 0.54 (0.13-2.33) | 0.411 |
|  | log-additive |  |  |  | 0.90 (0.64-1.27) | 0.559 |  |  | 0.88 (0.60-1.29) | 0.513 |
| rs4150658 | Genotype | AA | 216 | 209 | 1 |  | 191 | 188 | 1 |  |
|  |  | GA | 52 | 63 | 0.78 (0.51-1.18) | 0.243 | 43 | 43 | 0.98 (0.61-1.57) | 0.924 |
|  |  | GG | 3 | 3 | 0.98 (0.19-4.96) | 0.978 | 1 | 4 | 0.23 (0.03-2.11) | 0.194 |
|  | dominant | AA/GG+GA | 216/55 | 209/66 | 0.79 (0.52-1.19) | 0.255 | 191/44 | 188/47 | 0.91 (0.58-1.45) | 0.700 |
|  | recessive | GA+AA/GG | 268/3 | 272/3 | 1.03 (0.20-5.22) | 0.970 | 234/1 | 231/4 | 0.23 (0.03-2.11) | 0.195 |
|  | log-additive |  |  |  | 0.82 (0.56-1.19) | 0.295 |  |  | 0.86 (0.56-1.32) | 0.489 |
|  |  |  | **Males** | | | | **Females** | | | |
| rs11720298 | Genotype | AA | 204 | 197 | 1 |  | 82 | 71 | 1 |  |
|  |  | GA | 122 | 127 | 0.93 (0.68-1.27) | 0.642 | 63 | 68 | 0.8 0(0.50-1.28) | 0.355 |
|  |  | GG | 24 | 29 | 0.80 (0.45-1.42) | 0.445 | 11 | 17 | 0.56 (0.25-1.28) | 0.167 |
|  | dominant | AA/GG+GA | 204/146 | 197/156 | 0.90 (0.67-1.22) | 0.508 | 82/74 | 71/85 | 0.75 (0.48-1.18) | 0.213 |
|  | recessive | GA+AA/GG | 326/24 | 324/29 | 0.82 (0.47-1.45) | 0.497 | 145/11 | 139/17 | 0.62 (0.28-1.37) | 0.238 |
|  | log-additive |  |  |  | 0.91 (0.72-1.15) | 0.423 |  |  | 0.77 (0.55-1.09) | 0.138 |
| rs4687721 | Genotype | AA | 303 | 306 | 1 |  | 132 | 132 | 1 |  |
|  |  | GA | 46 | 45 | 1.03 (0.66-1.60) | 0.887 | 22 | 24 | 0.92 (0.49-1.72) | 0.788 |
|  |  | GG | 1 | 2 | 0.50 (0.05-5.59) | 0.577 | 2 | 1 | 2.04 (0.18-23.13) | 0.565 |
|  | dominant | AA/GG+GA | 303/47 | 306/47 | 1.01 (0.65-1.56) | 0.964 | 132/24 | 132/25 | 0.96 (0.52-1.77) | 0.898 |
|  | recessive | GA+AA/GG | 349/1 | 351/2 | 0.50 (0.05-5.57) | 0.575 | 154/2 | 156/1 | 2.07 (0.18-23.4) | 0.558 |
|  | log-additive |  |  |  | 0.99 (0.65-1.49) | 0.951 |  |  | 1.01 (0.58-1.76) | 0.977 |
| rs4687592 | Genotype | CC | 152 | 157 | 1 |  | 74 | 82 | 1 |  |
|  |  | TC | 153 | 149 | 1.06 (0.77-1.46) | 0.715 | 63 | 61 | 1.15 (0.71-1.84) | 0.575 |
|  |  | TT | 45 | 47 | 0.99 (0.62-1.58) | 0.963 | 19 | 14 | 1.51 (0.70-3.22) | 0.292 |
|  | dominant | CC/TT+TC | 152/198 | 157/196 | 1.04 (0.77-1.41) | 0.779 | 74/82 | 82/75 | 1.21 (0.78-1.89) | 0.398 |
|  | recessive | TC+CC/TT | 305/45 | 306/47 | 0.96 (0.62-1.49) | 0.857 | 137/19 | 143/14 | 1.42 (0.68-2.94) | 0.350 |
|  | log-additive |  |  |  | 1.01 (0.82-1.26) | 0.909 |  |  | 1.20 (0.86-1.67) | 0.289 |
| rs9864693 | Genotype | GG | 100 | 116 | 1 |  | 43 | 55 | 1 |  |
|  |  | GC | 187 | 168 | 1.29 (0.92-1.82) | 0.138 | 88 | 79 | 1.43 (0.86-2.36) | 0.167 |
|  |  | CC | 63 | 69 | 1.06 (0.69-1.64) | 0.795 | 25 | 23 | 1.39 (0.70-2.79) | 0.351 |
|  | dominant | GG/GC+CC | 100/250 | 116/237 | 1.22 (0.89-1.69) | 0.217 | 43/113 | 55/102 | 1.42 (0.88-2.30) | 0.155 |
|  | recessive | GG+GC/CC | 287/63 | 284/69 | 0.90 (0.62-1.32) | 0.599 | 131/25 | 134/23 | 1.11 (0.60-2.06) | 0.737 |
|  | log-additive |  |  |  | 1.06 (0.86-1.31) | 0.600 |  |  | 1.22 (0.87-1.71) | 0.240 |
| rs4150530 | Genotype | GG | 277 | 272 | 1 |  | 132 | 127 | 1 |  |
|  |  | TG | 70 | 74 | 0.93 (0.64-1.34) | 0.696 | 24 | 30 | / | / |
|  |  | TT | 3 | 7 | 0.42 (0.11-1.64) | 0.213 | 0 | 0 | / | / |
|  | dominant | GG/TT+TG | 277/73 | 272/81 | 0.89 (0.62-1.27) | 0.504 | 132/24 | 127/30 | 0.77 (0.43-1.39) | 0.384 |
|  | recessive | TG+GG/TT | 347/3 | 346/7 | 0.43 (0.11-1.67) | 0.220 | 156/0 | 157/0 | / | / |
|  | log-additive |  |  |  | 0.86 (0.62-1.19) | 0.350 |  |  | 0.77 (0.43-1.39) | 0.384 |
| rs3802967 | Genotype | CC | 107 | 90 | 1 |  | 46 | 43 | 1 |  |
|  |  | TC | 163 | 181 | 0.76 (0.53-1.08) | 0.120 | 78 | 82 | 0.89 (0.53-1.49) | 0.658 |
|  |  | TT | 80 | 82 | 0.82 (0.54-1.24) | 0.350 | 32 | 32 | 0.94 (0.49-1.78) | 0.838 |
|  | dominant | CC/TT+TC | 107/243 | 90/263 | 0.78 (0.56-1.08) | 0.133 | 46/110 | 43/114 | 0.90 (0.55-1.48) | 0.682 |
|  | recessive | TC+CC/TT | 270/80 | 271/82 | 0.98 (0.69-1.39) | 0.906 | 124/32 | 125/32 | 1.01 (0.58-1.75) | 0.976 |
|  | log-additive |  |  |  | 0.90 (0.73-1.11) | 0.310 |  |  | 0.96 (0.70-1.32) | 0.803 |
| rs4150606 | Genotype | AA | 256 | 254 | 1 |  | 123 | 121 | 1 |  |
|  |  | CA | 87 | 88 | 0.98 (0.70-1.38) | 0.914 | 32 | 35 | 0.90 (0.52-1.54) | 0.699 |
|  |  | CC | 7 | 11 | 0.63 (0.24-1.65) | 0.349 | 1 | 1 | 0.99 (0.06-15.96) | 0.992 |
|  | dominant | AA/CC+CA | 256/94 | 254/99 | 0.94 (0.68-1.31) | 0.725 | 123/33 | 121/36 | 0.90 (0.53-1.54) | 0.703 |
|  | recessive | CA+AA/CC | 343/7 | 342/11 | 0.63 (0.24-1.66) | 0.352 | 155/1 | 156/1 | 1.01 (0.06-16.28) | 0.995 |
|  | log-additive |  |  |  | 0.92 (0.69-1.22) | 0.551 |  |  | 0.91 (0.55-1.52) | 0.716 |
| rs4150658 | Genotype | AA | 275 | 270 | 1 |  | 132 | 127 | 1 |  |
|  |  | GA | 71 | 76 | 0.92 (0.64-1.32) | 0.644 | 24 | 30 | / | / |
|  |  | GG | 4 | 7 | 0.56 (0.16-1.94) | 0.361 | 0 | 0 | / | / |
|  | dominant | AA/GG+GA | 275/75 | 270/83 | 0.89 (0.62-1.27) | 0.509 | 132/24 | 127/30 | 0.77 (0.43-1.39) | 0.384 |
|  | recessive | GA+AA/GG | 346/4 | 346/7 | 0.57 (0.17-1.97) | 0.375 | 156/0 | 157/0 | / | / |
|  | log-additive |  |  |  | 0.87 (0.63-1.20) | 0.403 |  |  | 0.77 (0.43-1.39) | 0.384 |
| rs4150667 | Genotype | CC | 156 | 167 | 1 |  | 69 | 71 | 1 |  |
|  |  | TC | 160 | 149 | 1.15 (0.84-1.57) | 0.381 | 71 | 73 | 1.00 (0.63-1.59) | 0.998 |
|  |  | TT | 34 | 37 | 0.98 (0.59-1.65) | 0.952 | 16 | 13 | 1.27 (0.57-2.84) | 0.560 |
|  | dominant | CC/TT+TC | 156/194 | 167/186 | 1.12 (0.83-1.50) | 0.465 | 69/87 | 71/86 | 1.04 (0.67-1.63) | 0.859 |
|  | recessive | TC+CC/TT | 316/34 | 316/37 | 0.92 (0.56-1.50) | 0.736 | 140/16 | 144/13 | 1.27 (0.59-2.75) | 0.542 |
|  | log-additive |  |  |  | 1.05 (0.84-1.31) | 0.690 |  |  | 1.08 (0.76-1.52) | 0.681 |
|  |  |  | **Smokers** | | | | **Non-smokers** | | | |
| rs4687721 | Genotype | AA | 209 | 91 | 1 |  | 139 | 153 | 1 |  |
|  |  | GA | 32 | 16 | 0.85 (0.44-1.63) | 0.625 | 21 | 26 | 0.85 (0.44-1.61) | 0.609 |
|  |  | GG | 1 | 1 | 0.62 (0.03-11.34) | 0.745 | 1 | 1 | 1.15 (0.06-20.53) | 0.926 |
|  | dominant | AA/GG+GA | 209/33 | 91/17 | 0.84 (0.44-1.58) | 0.586 | 139/22 | 153/27 | 0.86 (0.45-1.61) | 0.630 |
|  | recessive | GA+AA/GG | 241/1 | 107/1 | 0.62 (0.03-11.4) | 0.749 | 160/1 | 179/1 | 1.17 (0.07-20.97) | 0.913 |
|  | log-additive |  |  |  | 0.84 (0.46-1.52) | 0.564 |  |  | 0.88 (0.48-1.59) | 0.667 |
| rs4687592 | Genotype | CC | 105 | 50 | 1 |  | 72 | 85 | 1 |  |
|  |  | TC | 105 | 45 | 1.12 (0.69-1.83) | 0.639 | 70 | 81 | 1.15 (0.72-1.84) | 0.563 |
|  |  | TT | 32 | 13 | 1.18 (0.57-2.44) | 0.665 | 19 | 14 | 1.46 (0.66-3.21) | 0.346 |
|  | dominant | CC/TT+TC | 105/137 | 50/58 | 1.14 (0.72-1.80) | 0.587 | 72/89 | 85/95 | 1.20 (0.77-1.88) | 0.421 |
|  | recessive | TC+CC/TT | 210/32 | 95/13 | 1.11 (0.56-2.22) | 0.767 | 142/19 | 166/14 | 1.37 (0.64-2.91) | 0.417 |
|  | log-additive |  |  |  | 1.10 (0.78-1.53) | 0.591 |  |  | 1.19 (0.84-1.67) | 0.327 |
| rs9864693 | Genotype | GG | 74 | 37 | 1 |  | 4 | 60 | 1 |  |
|  |  | GC | 124 | 51 | 1.23 (0.73-2.05) | 0.439 | 91 | 94 | 1.44 (0.87-2.39) | 0.159 |
|  |  | CC | 44 | 20 | 1.11 (0.57-2.16) | 0.756 | 26 | 26 | 1.43 (0.71-2.87) | 0.315 |
|  | dominant | GG/GC+CC | 74/168 | 37/71 | 1.19 (0.73-1.94) | 0.476 | 44/117 | 60/120 | 1.44 (0.88-2.34) | 0.144 |
|  | recessive | GG+GC/CC | 198/44 | 88/20 | 0.98 (0.55-1.77) | 0.952 | 135/26 | 154/26 | 1.13 (0.61-2.09) | 0.694 |
|  | log-additive |  |  |  | 1.08 (0.78-1.50) | 0.657 |  |  | 1.24 (0.88-1.73) | 0.217 |
| rs4150530 | Genotype | GG | 190 | 88 | 1 |  | 136 | 140 | 1 |  |
|  |  | TG | 50 | 17 | 1.35 (0.73-2.48) | 0.334 | 25 | 38 | 0.79 (0.44-1.42) | 0.433 |
|  |  | TT | 2 | 3 | 0.30 (0.05-1.83) | 0.193 | 0 | 2 | / | / |
|  | dominant | GG/TT+TG | 190/52 | 88/20 | 1.19 (0.67-2.12) | 0.553 | 136/25 | 140/40 | 0.76 (0.43-1.36) | 0.356 |
|  | recessive | TG+GG/TT | 240/2 | 105/3 | 0.28 (0.05-1.73) | 0.172 | 161/0 | 178/2 | / | / |
|  | log-additive |  |  |  | 1.04 (0.62-1.74) | 0.880 |  |  | 0.74 (0.42-1.29) | 0.287 |
| rs3802967 | Genotype | CC | 74 | 25 | 1 |  | 49 | 49 | 1 |  |
|  |  | TC | 110 | 63 | 0.60 (0.35-1.04) | 0.070 | 79 | 86 | 0.89 (0.53-1.50) | 0.663 |
|  |  | TT | 58 | 20 | 1.01 (0.51-1.99) | 0.987 | 33 | 45 | 0.83 (0.44-1.55) | 0.558 |
|  | dominant | CC/TT+TC | 74/168 | 25/83 | 0.70 (0.41-1.18) | 0.180 | 49/112 | 49/131 | 0.87 (0.53-1.42) | 0.580 |
|  | recessive | TC+CC/TT | 184/58 | 88/20 | 1.41 (0.80-2.49) | 0.240 | 128/33 | 135/45 | 0.89 (0.52-1.52) | 0.675 |
|  | log-additive |  |  |  | 0.97 (0.71-1.34) | 0.874 |  |  | 0.91 (0.67-1.24) | 0.551 |
| rs4150606 | Genotype | AA | 181 | 81 | 1 |  | 125 | 132 | 1 |  |
|  |  | CA | 56 | 22 | 1.11 (0.63-1.95) | 0.716 | 34 | 45 | 0.93 (0.55-1.58) | 0.790 |
|  |  | CC | 5 | 5 | 0.47 (0.13-1.69) | 0.247 | 2 | 3 | 1.07 (0.17-6.85) | 0.942 |
|  | dominant | AA/CC+CA | 181/61 | 81/27 | 0.99 (0.59-1.68) | 0.980 | 125/36 | 132/48 | 0.94 (0.56-1.58) | 0.809 |
|  | recessive | CA+AA/CC | 237/5 | 103/5 | 0.46 (0.13-1.64) | 0.231 | 159/2 | 177/3 | 1.09 (0.17-6.95) | 0.925 |
|  | log-additive |  |  |  | 0.91 (0.58-1.41) | 0.672 |  |  | 0.95 (0.59-1.54) | 0.844 |
| rs4150658 | Genotype | AA | 189 | 87 | 1 |  | 135 | 139 | 1 |  |
|  |  | GA | 50 | 18 | 1.27 (0.70-2.30) | 0.441 | 26 | 39 | 0.82 (0.46-1.46) | 0.498 |
|  |  | GG | 3 | 3 | 0.45 (0.09-2.27) | 0.332 | 0 | 2 | / | / |
|  | dominant | AA/GG+GA | 189/53 | 87/21 | 1.15 (0.65-2.03) | 0.635 | 135/26 | 139/41 | 0.79 (0.45-1.4) | 0.416 |
|  | recessive | GA+AA/GG | 239/3 | 105/3 | 0.43 (0.08-2.16) | 0.304 | 161/0 | 178/2 | / | / |
|  | log-additive |  |  |  | 1.03 (0.63-1.70) | 0.902 |  |  | 0.76 (0.44-1.32) | 0.336 |
| rs4150667 | Genotype | CC | 113 | 49 | 1 |  | 66 | 92 | 1 |  |
|  |  | TC | 1006 | 47 | 0.97 (0.60-1.57) | 0.891 | 80 | 75 | 1.39 (0.87-2.22) | 0.164 |
|  |  | TT | 23 | 12 | 0.80 (0.37-1.74) | 0.578 | 15 | 13 | 1.50 (0.65-3.46) | 0.345 |
|  | dominant | CC/TT+TC | 113/129 | 49/59 | 0.93 (0.59-1.47) | 0.767 | 66/95 | 92/88 | 1.41 (0.90-2.20) | 0.134 |
|  | recessive | TC+CC/TT | 219/23 | 96/12 | 0.82 (0.39-1.71) | 0.589 | 146/15 | 167/13 | 1.27 (0.57-2.84) | 0.562 |
|  | log-additive |  |  |  | 0.92 (0.65-1.30) | 0.637 |  |  | 1.29 (0.91-1.84) | 0.152 |
|  |  |  | **Drinkers** | | | | **Non-drinkers** | | | |
| rs4687721 | Genotype | AA | 95 | 90 | 1 |  | 230 | 130 | 1 |  |
|  |  | GA | 14 | 12 | 1.07 (0.47-2.45) | 0.877 | 36 | 25 | 0.81 (0.46-1.41) | 0.454 |
|  |  | GG | 0 | 1 | / | / | 1 | 1 | 0.56 (0.03-9.01) | 0.682 |
|  | dominant | AA/GG+GA | 95/14 | 90/13 | 1.01 (0.45-2.28) | 0.986 | 230/37 | 130/26 | 0.80 (0.46-1.38) | 0.422 |
|  | recessive | GA+AA/GG | 109/0 | 102/1 | / | / | 266/1 | 155/1 | 0.58 (0.04-9.29) | 0.698 |
|  | log-additive |  |  |  | 0.96 (0.44-2.09) | 0.920 |  |  | 0.80 (0.48-1.35) | 0.403 |
| rs9864693 | Genotype | GG | 27 | 34 | 1 |  | 79 | 55 | 1 |  |
|  |  | GC | 56 | 52 | 1.37 (0.73-2.59) | 0.331 | 147 | 77 | 1.35 (0.87-2.11) | 0.181 |
|  |  | CC | 26 | 17 | 2.09 (0.93-4.69) | 0.074 | 41 | 24 | 1.21 (0.65-2.23) | 0.546 |
|  | dominant | GG/GC+CC | 27/82 | 34/69 | 1.54 (0.84-2.81) | 0.161 | 79/188 | 55/101 | 1.32 (0.86-2.01) | 0.200 |
|  | recessive | GG+GC/CC | 83/26 | 86/17 | 1.71 (0.85-3.43) | 0.132 | 226/41 | 132/24 | 1.00 (0.58-1.73) | 0.998 |
|  | log-additive |  |  |  | 1.44 (0.96-2.14) | 0.075 |  |  | 1.15 (0.85-1.55) | 0.370 |
| rs4150530 | Genotype | GG | 82 | 85 | 1 |  | 220 | 121 | 1 |  |
|  |  | TG | 26 | 14 | 1.89 (0.92-3.88) | 0.084 | 46 | 34 | 0.73 (0.44-1.21) | 0.220 |
|  |  | TT | 1 | 4 | 0.25 (0.03-2.29) | 0.220 | 1 | 1 | 0.52 (0.03-8.53) | 0.650 |
|  | dominant | GG/TT+TG | 82/27 | 85/18 | 1.52 (0.78-2.98) | 0.221 | 220/47 | 121/35 | 0.73 (0.44-1.19) | 0.205 |
|  | recessive | TG+GG/TT | 108/1 | 99/4 | 0.22 (0.02-2.02) | 0.181 | 266/1 | 155/1 | 0.57 (0.03-9.18) | 0.689 |
|  | log-additive |  |  |  | 1.19 (0.67-2.10) | 0.558 |  |  | 0.73 (0.45-1.18) | 0.197 |
| rs3802967 | Genotype | CC | 27 | 26 | 1 |  | 8 | 43 | 1 |  |
|  |  | TC | 51 | 52 | 0.98 (0.50-1.92) | 0.960 | 124 | 80 | 0.80 (0.50-1.28) | 0.359 |
|  |  | TT | 31 | 25 | 1.20 (0.56-2.55) | 0.639 | 61 | 33 | 0.94 (0.54-1.66) | 0.834 |
|  | dominant | CC/TT+TC | 27/82 | 26/77 | 1.06 (0.57-1.98) | 0.864 | 82/185 | 43/113 | 0.84 (0.54-1.31) | 0.449 |
|  | recessive | TC+CC/TT | 78/31 | 78/25 | 1.21 (0.65-2.25) | 0.543 | 206/61 | 123/33 | 1.08 (0.67-1.75) | 0.756 |
|  | log-additive |  |  |  | 1.10 (0.75-1.60) | 0.634 |  |  | 0.96 (0.73-1.26) | 0.762 |
| rs4150606 | Genotype | AA | 75 | 78 | 1 |  | 207 | 114 | 1 |  |
|  |  | CA | 32 | 19 | 1.74 (0.91-3.34) | 0.096 | 55 | 41 | 0.73 (0.46-1.17) | 0.197 |
|  |  | CC | 2 | 6 | 0.33 (0.06-1.71) | 0.187 | 5 | 1 | 2.55 (0.29-22.21) | 0.396 |
|  | dominant | AA/CC+CA | 75/34 | 78/25 | 1.40 (0.76-2.57) | 0.279 | 207/60 | 114/42 | 0.78 (0.49-1.23) | 0.286 |
|  | recessive | CA+AA/CC | 107/2 | 97/6 | 0.29 (0.06-1.48) | 0.136 | 262/5 | 155/1 | 2.76 (0.32-23.94) | 0.357 |
|  | log-additive |  |  |  | 1.09 (0.66-1.80) | 0.731 |  |  | 0.85 (0.56-1.30) | 0.463 |
| rs4150658 | Genotype | AA | 80 | 84 | 1 |  | 220 | 120 | 1 |  |
|  |  | GA | 27 | 15 | 1.85 (0.92-3.75) | 0.086 | 46 | 35 | 0.70 (0.43-1.16) | 0.164 |
|  |  | GG | 2 | 4 | 0.51 (0.09-2.85) | 0.441 | 1 | 1 | 0.52 (0.03-8.43) | 0.644 |
|  | dominant | AA/GG+GA | 80/29 | 84/19 | 1.57 (0.81-3.03) | 0.180 | 220/47 | 120/36 | 0.70 (0.43-1.14) | 0.152 |
|  | recessive | GA+AA/GG | 107/2 | 99/4 | 0.45 (0.08-2.50) | 0.359 | 266/1 | 155/1 | 0.57 (0.03-9.18) | 0.689 |
|  | log-additive |  |  |  | 1.26 (0.72-2.19) | 0.414 |  |  | 0.70 (0.44-1.13) | 0.148 |
| rs4150667 | Genotype | CC | 55 | 55 | 1 |  | 112 | 71 | 1 |  |
|  |  | TC | 43 | 40 | 1.09 (0.62-1.94) | 0.763 | 133 | 73 | 1.17 (0.77-1.77) | 0.454 |
|  |  | TT | 11 | 8 | 1.37 (0.51-3.68) | 0.533 | 22 | 12 | 1.18 (0.55-2.54) | 0.669 |
|  | dominant | CC/TT+TC | 55/54 | 55/48 | 1.14 (0.66-1.96) | 0.639 | 112/155 | 71/85 | 1.17 (0.79-1.75) | 0.434 |
|  | recessive | TC+CC/TT | 98/11 | 95/8 | 1.32 (0.51-3.43) | 0.571 | 245/22 | 144/12 | 1.09 (0.52-2.27) | 0.824 |
|  | log-additive |  |  |  | 1.14 (0.75-1.73) | 0.542 |  |  | 1.12 (0.82-1.55) | 0.473 |
|  |  |  | **BMI > 24 kg/m^2^** | | | | **BMI ≤ 24 kg/m^2^** | | | |
| rs11720298 | Genotype | AA | 50 | 93 | 1 |  | 70 | 78 | 1 |  |
|  |  | GA | 28 | 76 | 0.65 (0.37-1.14) | 0.136 | 54 | 43 | 1.46 (0.87-2.46) | 0.155 |
|  |  | GG | 3 | 12 | 0.45 (0.12-1.68) | 0.233 | 9 | 16 | 0.66 (0.27-1.61) | 0.360 |
|  | dominant | AA/GG+GA | 50/31 | 93/88 | 0.62 (0.36-1.07) | 0.089 | 70/63 | 78/59 | 1.25 (0.77-2.04) | 0.372 |
|  | recessive | GA+AA/GG | 78/3 | 169/12 | 0.53 (0.14-1.96) | 0.342 | 124/9 | 121/16 | 0.56 (0.24-1.34) | 0.195 |
|  | log-additive |  |  |  | 0.66 (0.41-1.05) | 0.077 |  |  | 1.02 (0.70-1.48) | 0.921 |
| rs4687721 | Genotype | AA | 71 | 158 | 1 |  | 116 | 114 | 1 |  |
|  |  | GA | 10 | 22 | 0.93 (0.41-2.09) | 0.858 | 17 | 23 | 0.70 (0.35-1.38) | 0.299 |
|  |  | GG | 0 | 1 | / | / | 0 | 1 | / | / |
|  | dominant | AA/GG+GA | 71/10 | 158/23 | 0.89 (0.40-2.00) | 0.782 | 116/17 | 114/24 | 0.66 (0.34-1.31) | 0.238 |
|  | recessive | GA+AA/GG | 81/0 | 180/1 | / | / | 133/0 | 137/1 | / | / |
|  | log-additive |  |  |  | 0.86 (0.39-1.88) | 0.704 |  |  | 0.64 (0.33-1.24) | 0.190 |
| rs4687592 | Genotype | CC | 44 | 86 | 1 |  | 46 | 63 | 1 |  |
|  |  | TC | 29 | 77 | 0.69 (0.39-1.23) | 0.210 | 70 | 60 | 1.53 (0.91-2.56) | 0.109 |
|  |  | TT | 8 | 18 | 0.87 (0.34-2.18) | 0.763 | 17 | 15 | 1.49 (0.67-3.31) | 0.326 |
|  | dominant | CC/TT+TC | 44/37 | 86/95 | 0.73 (0.43-1.24) | 0.241 | 46/87 | 63/75 | 1.52 (0.93-2.49) | 0.097 |
|  | recessive | TC+CC/TT | 73/8 | 163/18 | 1.02 (0.42-2.48) | 0.970 | 116/17 | 123/15 | 1.18 (0.56-2.48) | 0.661 |
|  | log-additive |  |  |  | 0.83 (0.55-1.26) | 0.383 |  |  | 1.31 (0.91-1.89) | 0.151 |
| rs9864693 | Genotype | GG | 29 | 62 | 1 |  | 29 | 41 | 1 |  |
|  |  | GC | 42 | 94 | 0.89 (0.50-1.59) | 0.693 | 78 | 70 | 1.52 (0.84-2.73) | 0.163 |
|  |  | CC | 10 | 25 | 0.82 (0.34-1.95) | 0.650 | 26 | 27 | 1.25 (0.6-2.59) | 0.550 |
|  | dominant | GG/GC+CC | 29/52 | 62/119 | 0.87 (0.50-1.53) | 0.637 | 29/104 | 41/97 | 1.44 (0.82-2.52) | 0.202 |
|  | recessive | GG+GC/CC | 71/10 | 156/25 | 0.88 (0.40-1.95) | 0.748 | 107/26 | 111/27 | 0.94 (0.51-1.73) | 0.842 |
|  | log-additive |  |  |  | 0.90 (0.60-1.35) | 0.613 |  |  | 1.14 (0.79-1.64) | 0.478 |
| rs4150530 | Genotype | GG | 64 | 147 | 1 |  | 111 | 111 | 1 |  |
|  |  | TG | 17 | 34 | / | / | 21 | 26 | 0.76 (0.40-1.44) | 0.393 |
|  |  | TT | 0 | 0 | / | / | 1 | 1 | 1.07 (0.06-17.50) | 0.965 |
|  | dominant | GG/TT+TG | 64/17 | 147/34 | 1.22 (0.63-2.37) | 0.555 | 111/22 | 111/27 | 0.77 (0.41-1.44) | 0.410 |
|  | recessive | TG+GG/TT | 81/0 | 181/0 | / | / | 132/1 | 137/1 | 1.12 (0.07-18.32) | 0.938 |
|  | log-additive |  |  |  | 1.22 (0.63-2.37) | 0.555 |  |  | 0.80 (0.44-1.44) | 0.448 |
| rs3802967 | Genotype | CC | 29 | 48 | 1 |  | 34 | 35 | 1 |  |
|  |  | TC | 36 | 98 | 0.63 (0.34-1.16) | 0.135 | 66 | 71 | 0.95 (0.53-1.71) | 0.873 |
|  |  | TT | 16 | 35 | 0.85 (0.40-1.83) | 0.680 | 33 | 32 | 1.08 (0.54-2.13) | 0.830 |
|  | dominant | CC/TT+TC | 29/52 | 48/133 | 0.68 (0.39-1.21) | 0.192 | 34/99 | 35/103 | 0.99 (0.57-1.72) | 0.977 |
|  | recessive | TC+CC/TT | 65/16 | 146/35 | 1.14 (0.58-2.23) | 0.709 | 100/33 | 106/32 | 1.11 (0.63-1.95) | 0.711 |
|  | log-additive |  |  |  | 0.88 (0.59-1.30) | 0.513 |  |  | 1.04 (0.74-1.46) | 0.836 |
| rs4150606 | Genotype | AA | 62 | 138 | 1 |  | 103 | 104 | 1 |  |
|  |  | CA | 17 | 41 | 0.97 (0.50-1.85) | 0.918 | 28 | 32 | 0.84 (0.47-1.50) | 0.547 |
|  |  | CC | 2 | 2 | 2.55 (0.35-18.72) | 0.356 | 2 | 2 | 0.97 (0.13-7.07) | 0.979 |
|  | dominant | AA/CC+CA | 62/19 | 138/43 | 1.04 (0.55-1.94) | 0.911 | 103/30 | 104/34 | 0.84 (0.48-1.49) | 0.559 |
|  | recessive | CA+AA/CC | 79/2 | 179/2 | 2.58 (0.35-18.76) | 0.351 | 131/2 | 136/2 | 1.02 (0.14-7.34) | 0.988 |
|  | log-additive |  |  |  | 1.11 (0.63-1.95) | 0.724 |  |  | 0.87 (0.52-1.46) | 0.598 |
| rs4150658 | Genotype | AA | 64 | 146 | 1 |  | 111 | 110 | 1 |  |
|  |  | GA | 17 | 35 | / | / | 21 | 27 | 0.72 (0.38-1.37) | 0.316 |
|  |  | GG | 0 | 0 | / | / | 1 | 1 | 1.05 (0.06-17.32) | 0.971 |
|  | dominant | AA/GG+GA | 64/17 | 146/35 | 1.18 (0.61-2.27) | 0.632 | 111/22 | 110/28 | 0.73 (0.39-1.37) | 0.331 |
|  | recessive | GA+AA/GG | 81/0 | 181/0 | / | / | 132/1 | 137/1 | 1.12 (0.07-18.32) | 0.938 |
|  | log-additive |  |  |  | 1.18 (0.61-2.27) | 0.632 |  |  | 0.76 (0.42-1.38) | 0.369 |
|  |  |  | **Lung adenocarcinoma** | | | | **Lung squamous cell carcinoma** | | | |
| rs11720298 | Genotype | AA | 119 | 268 | 1 |  | 95 | 268 | 1 |  |
|  |  | GA | 79 | 195 | 0.87 (0.62-1.23) | 0.434 | 67 | 195 | 1.02 (0.70-1.48) | 0.918 |
|  |  | GG | 14 | 46 | 0.66 (0.35-1.25) | 0.197 | 12 | 46 | 0.73 (0.36-1.46) | 0.369 |
|  | dominant | AA/GG+GA | 119/93 | 268/241 | 0.83 (0.60-1.15) | 0.265 | 95/79 | 268/241 | 0.96 (0.68-1.37) | 0.834 |
|  | recessive | GA+AA/GG | 198/14 | 463/46 | 0.69 (0.37-1.30) | 0.253 | 162/12 | 463/46 | 0.72 (0.37-1.42) | 0.344 |
|  | log-additive |  |  |  | 0.84 (0.65-1.08) | 0.175 |  |  | 0.92 (0.70-1.22) | 0.567 |
| rs4687721 | Genotype | AA | 186 | 438 | 1 |  | 147 | 438 | 1 |  |
|  |  | GA | 25 | 69 | 0.83 (0.51-1.36) | 0.464 | 25 | 69 | 1.12 (0.68-1.85) | 0.661 |
|  |  | GG | 1 | 3 | 0.69 (0.07-6.80) | 0.747 | 2 | 3 | 2.73 (0.42-17.92) | 0.296 |
|  | dominant | AA/GG+GA | 186/26 | 438/72 | 0.83 (0.51-1.34) | 0.438 | 147/27 | 438/72 | 1.17 (0.72-1.92) | 0.523 |
|  | recessive | GA+AA/GG | 211/1 | 507/3 | 0.70 (0.07-6.95) | 0.762 | 172/2 | 507/3 | 2.69 (0.41-17.61) | 0.303 |
|  | log-additive |  |  |  | 0.83 (0.53-1.31) | 0.428 |  |  | 1.21 (0.77-1.91) | 0.410 |
| rs4687592 | Genotype | CC | 90 | 239 | 1 |  | 77 | 239 | 1 |  |
|  |  | TC | 92 | 210 | 1.22 (0.86-1.73) | 0.276 | 74 | 210 | 1.03 (0.71-1.51) | 0.864 |
|  |  | TT | 30 | 61 | 1.40 (0.84-2.32) | 0.196 | 23 | 61 | 1.07 (0.61-1.86) | 0.814 |
|  | dominant | CC/TT+TC | 90/122 | 239/271 | 1.26 (0.90-1.74) | 0.176 | 77/97 | 239/271 | 1.04 (0.73-1.48) | 0.821 |
|  | recessive | TC+CC/TT | 182/30 | 449/61 | 1.27 (0.79-2.04) | 0.325 | 151/23 | 449/61 | 1.05 (0.62-1.77) | 0.850 |
|  | log-additive |  |  |  | 1.19 (0.94-1.51) | 0.145 |  |  | 1.03 (0.80-1.33) | 0.799 |
| rs9864693 | Genotype | GG | 57 | 171 | 1 |  | 49 | 171 | 1 |  |
|  |  | GC | 115 | 247 | 1.41 (0.97-2.06) | 0.073 | 91 | 247 | 1.27 (0.84-1.90) | 0.252 |
|  |  | CC | 40 | 92 | 1.37 (0.84-2.22) | 0.204 | 34 | 92 | 1.24 (0.74-2.07) | 0.416 |
|  | dominant | GG/GC+CC | 57/155 | 171/339 | 1.40 (0.98-2.01) | 0.066 | 49/125 | 171/339 | 1.26 (0.86-1.85) | 0.240 |
|  | recessive | GG+GC/CC | 172/40 | 418/92 | 1.10 (0.73-1.66) | 0.658 | 140/34 | 418/92 | 1.07 (0.69-1.67) | 0.770 |
|  | log-additive |  |  |  | 1.19 (0.94-1.51) | 0.139 |  |  | 1.13 (0.88-1.45) | 0.344 |
| rs4150530 | Genotype | GG | 166 | 399 | 1 |  | 141 | 399 | 1 |  |
|  |  | TG | 43 | 104 | 1.04 (0.69-1.55) | 0.861 | 33 | 104 | 0.85 (0.55-1.33) | 0.482 |
|  |  | TT | 3 | 7 | 1.26 (0.32-4.96) | 0.743 | 0 | 7 | / | / |
|  | dominant | GG/TT+TG | 166/46 | 399/111 | 1.05 (0.71-1.55) | 0.811 | 141/33 | 399/111 | 0.79 (0.51-1.23) | 0.300 |
|  | recessive | TG+GG/TT | 209/3 | 503/7 | 1.25 (0.32-4.91) | 0.751 | 174/0 | 503/7 | / | / |
|  | log-additive |  |  |  | 1.06 (0.74-1.51) | 0.765 |  |  | 0.75 (0.49-1.14) | 0.175 |
| rs4150606 | Genotype | AA | 152 | 375 | 1 |  | 130 | 375 | 1 |  |
|  |  | CA | 55 | 123 | 1.14 (0.79-1.66) | 0.480 | 43 | 123 | 0.96 (0.64-1.44) | 0.830 |
|  |  | CC | 5 | 12 | 1.23 (0.42-3.58) | 0.704 | 1 | 12 | 0.20 (0.03-1.59) | 0.129 |
|  | dominant | AA/CC+CA | 152/60 | 375/135 | 1.15 (0.80-1.65) | 0.447 | 130/44 | 375/135 | 0.88 (0.59-1.32) | 0.543 |
|  | recessive | CA+AA/CC | 207/5 | 498/12 | 1.19 (0.41-3.43) | 0.754 | 173/1 | 498/12 | 0.21 (0.03-1.60) | 0.131 |
|  | log-additive |  |  |  | 1.13 (0.82-1.56) | 0.443 |  |  | 0.83 (0.57-1.19) | 0.308 |
| rs4150658 | Genotype | AA | 164 | 397 | 1 |  | 141 | 397 | 1 |  |
|  |  | GA | 44 | 106 | 1.05 (0.71-1.57) | 0.797 | 33 | 106 | 0.83 (0.53-1.29) | 0.406 |
|  |  | GG | 4 | 7 | 1.70 (0.49-5.94) | 0.405 | 0 | 7 | / | / |
|  | dominant | AA/GG+GA | 164/48 | 397/113 | 1.09 (0.74-1.61) | 0.666 | 141/33 | 397/113 | 0.77 (0.5-1.20) | 0.247 |
|  | recessive | GA+AA/GG | 208/4 | 503/7 | 1.68 (0.48-5.85) | 0.414 | 174/0 | 503/7 | / | / |
|  | log-additive |  |  |  | 1.12 (0.79-1.58) | 0.544 |  |  | 0.73 (0.48-1.11) | 0.142 |
| rs4150667 | Genotype | CC | 94 | 238 | 1 |  | 75 | 238 | 1 |  |
|  |  | TC | 97 | 222 | 1.09 (0.78-1.54) | 0.611 | 79 | 222 | 1.11 (0.77-1.62) | 0.568 |
|  |  | TT | 21 | 50 | 1.06 (0.60-1.87) | 0.842 | 20 | 50 | 1.23 (0.68-2.23) | 0.488 |
|  | dominant | CC/TT+TC | 94/118 | 238/272 | 1.09 (0.79-1.50) | 0.617 | 75/99 | 238/272 | 1.14 (0.80-1.62) | 0.479 |
|  | recessive | TC+CC/TT | 191/21 | 460/50 | 1.01 (0.59-1.74) | 0.960 | 154/20 | 460/50 | 1.17 (0.67-2.05) | 0.587 |
|  | log-additive |  |  |  | 1.05 (0.82-1.35) | 0.687 |  |  | 1.11 (0.85-1.45) | 0.432 |

SNP, single nucleotide polymorphism; OR, odds ratio; 95% CI, 95% confidence interval.

*p* values were calculated by logistic regression analysis with adjustments for age and gender.

/ showed no results.

Suppl_Table 4. The associations between *RAD54L2* and *GTF2H1* polymorphisms and the stage and metastasis of lung cancer

| SNP | Model | Genotype | Stage III-IV | Stage I-II | OR (95% CI) | *p*-value | Metastasis | Non-metastasis | OR (95% CI) | *p*-value |
| --- | --- | --- | --- | --- | --- | --- | --- | --- | --- | --- |
| rs11720298 | Genotype | AA | 156 | 57 | 1 |  | 154 | 61 | 1 |  |
|  |  | GA | 109 | 28 | 1.38 (0.82-2.31) | 0.226 | 97 | 34 | 1.12 (0.69-1.83) | 0.652 |
|  |  | GG | 21 | 8 | 0.96 (0.40-2.29) | 0.921 | 18 | 8 | 0.88 (0.36-2.13) | 0.770 |
|  | dominant | AA/GG+GA | 156/130 | 57/36 | 1.28 (0.79-2.08) | 0.310 | 154/115 | 61/42 | 1.07 (0.68-1.70) | 0.764 |
|  | recessive | GA+AA/GG | 265/21 | 85/8 | 0.85 (0.36-2.00) | 0.710 | 251/18 | 95/8 | 0.84 (0.35-2.00) | 0.693 |
|  | log-additive |  |  |  | 1.13 (0.77-1.65) | 0.525 |  |  | 1.01 (0.70-1.46) | 0.939 |
| rs4687721 | Genotype | AA | 247 | 78 | 1 |  | 226 | 88 | 1 |  |
|  |  | GA | 38 | 14 | 0.88 (0.45-1.71) | 0.695 | 42 | 14 | 1.17 (0.61-2.26) | 0.632 |
|  |  | GG | 1 | 1 | 0.30 (0.02-4.92) | 0.395 | 1 | 1 | 0.38 (0.02-6.22) | 0.497 |
|  | dominant | AA/GG+GA | 247/39 | 78/15 | 0.84 (0.44-1.60) | 0.589 | 226/43 | 88/15 | 1.12 (0.59-2.12) | 0.727 |
|  | recessive | GA+AA/GG | 285/1 | 92/1 | 0.30 (0.02-5.01) | 0.403 | 268/1 | 102/1 | 0.37 (0.02-6.07) | 0.487 |
|  | log-additive |  |  |  | 0.81 (0.44-1.49) | 0.495 |  |  | 1.06 (0.58-1.94) | 0.846 |
| rs4687592 | Genotype | CC | 136 | 40 | 1 |  | 121 | 46 | 1 |  |
|  |  | TC | 115 | 40 | 0.86 (0.52-1.42) | 0.555 | 110 | 43 | 0.97 (0.59-1.59) | 0.905 |
|  |  | TT | 35 | 13 | 0.79 (0.38-1.65) | 0.532 | 38 | 14 | 1.02 (0.51-2.07) | 0.949 |
|  | dominant | CC/TT+TC | 136/150 | 40/53 | 0.84 (0.52-1.35) | 0.477 | 121/148 | 46/57 | 0.98 (0.62-1.55) | 0.944 |
|  | recessive | TC+CC/TT | 251/35 | 80/13 | 0.85 (0.43-1.70) | 0.649 | 231/38 | 89/14 | 1.04 (0.54-2.01) | 0.912 |
|  | log-additive |  |  |  | 0.88 (0.63-1.24) | 0.464 |  |  | 1.00 (0.72-1.38) | 0.996 |
| rs9864693 | Genotype | GG | 90 | 27 | 1 |  | 75 | 32 | 1 |  |
|  |  | GC | 149 | 48 | 0.93 (0.54-1.60) | 0.787 | 142 | 51 | 1.17 (0.69-1.97) | 0.569 |
|  |  | CC | 47 | 18 | 0.80 (0.40-1.60) | 0.527 | 52 | 20 | 1.10 (0.57-2.14) | 0.774 |
|  | dominant | GG/GC+CC | 90/196 | 27/66 | 0.89 (0.53-1.50) | 0.667 | 75/194 | 32/71 | 1.15 (0.70-1.89) | 0.588 |
|  | recessive | GG+GC/CC | 239/47 | 75/18 | 0.84 (0.46-1.53) | 0.565 | 217/52 | 83/20 | 1.00 (0.56-1.78) | 0.999 |
|  | log-additive |  |  |  | 0.90 (0.64-1.27) | 0.541 |  |  | 1.06 (0.76-1.48) | 0.721 |
| rs4150530 | Genotype | GG | 231 | 74 | 1 |  | 221 | 81 | 1 |  |
|  |  | TG | 52 | 19 | 0.93 (0.51-1.68) | 0.808 | 46 | 22 | 0.78 (0.44-1.38) | 0.393 |
|  |  | TT | 3 | 0 | / | / | 2 | 0 | / | / |
|  | dominant | GG/TT+TG | 231/55 | 74/19 | 0.99 (0.55-1.78) | 0.963 | 221/48 | 81/22 | 0.81 (0.46-1.44) | 0.478 |
|  | recessive | TG+GG/TT | 283/3 | 93/0 | / | / | 267/2 | 103/0 | / | / |
|  | log-additive |  |  |  | 1.06 (0.60-1.85) | 0.851 |  |  | 0.87 (0.50-1.50) | 0.607 |
| rs3802967 | Genotype | CC | 81 | 31 | 1 |  | 79 | 34 | 1 |  |
|  |  | TC | 141 | 36 | 1.50 (0.86-2.62) | 0.153 | 133 | 41 | 1.40 (0.82-2.39) | 0.217 |
|  |  | TT | 64 | 26 | 0.95 (0.51-1.77) | 0.873 | 57 | 28 | 0.88 (0.48-1.61) | 0.668 |
|  | dominant | CC/TT+TC | 81/205 | 31/62 | 1.27 (0.77-2.10) | 0.355 | 79/190 | 34/69 | 1.19 (0.73-1.93) | 0.491 |
|  | recessive | TC+CC/TT | 222/64 | 67/26 | 0.75 (0.44-1.28) | 0.292 | 212/57 | 75/28 | 0.72 (0.43-1.22) | 0.218 |
|  | log-additive |  |  |  | 0.99 (0.72-1.37) | 0.969 |  |  | 0.96 (0.70-1.31) | 0.782 |
| rs4150606 | Genotype | AA | 214 | 70 | 1 |  | 207 | 73 | 1 |  |
|  |  | CA | 65 | 22 | 1.01 (0.58-1.76) | 0.981 | 56 | 30 | 0.67 (0.40-1.12) | 0.124 |
|  |  | CC | 7 | 1 | 2.59 (0.31-21.48) | 0.379 | 6 | 0 | / | / |
|  | dominant | AA/CC+CA | 214/72 | 70/23 | 1.07 (0.62-1.85) | 0.798 | 207/62 | 73/30 | 0.74 (0.44-1.23) | 0.246 |
|  | recessive | CA+AA/CC | 279/7 | 92/1 | 2.58 (0.31-21.35) | 0.379 | 263/6 | 103/0 | / | / |
|  | log-additive |  |  |  | 1.13 (0.69-1.85) | 0.619 |  |  | 0.86 (0.54-1.38) | 0.531 |
| rs4150658 | Genotype | AA | 229 | 74 | 1 |  | 219 | 81 | 1 |  |
|  |  | GA | 53 | 19 | 0.96 (0.53-1.73) | 0.892 | 47 | 22 | 0.81 (0.46-1.42) | 0.458 |
|  |  | GG | 4 | 0 | / | / | 3 | 0 | / | / |
|  | dominant | AA/GG+GA | 229/57 | 74/19 | 1.04 (0.58-1.87) | 0.904 | 219/50 | 81/22 | 0.86 (0.49-1.51) | 0.597 |
|  | recessive | GA+AA/GG | 282/4 | 93/0 | / | / | 266/3 | 103/0 | / | / |
|  | log-additive |  |  |  | 1.12 (0.65-1.95) | 0.681 |  |  | 0.93 (0.55-1.59) | 0.797 |
| rs4150667 | Genotype | CC | 131 | 42 | 1 |  | 119 | 42 | 1 |  |
|  |  | TC | 127 | 43 | 0.93 (0.57-1.53) | 0.787 | 121 | 52 | 0.82 (0.51-1.33) | 0.429 |
|  |  | TT | 28 | 8 | 1.11 (0.47-2.63) | 0.817 | 29 | 9 | 1.14 (0.50-2.62) | 0.752 |
|  | dominant | CC/TT+TC | 131/155 | 42/51 | 0.96 (0.60-1.54) | 0.871 | 119/150 | 42/61 | 0.87 (0.55-1.38) | 0.558 |
|  | recessive | TC+CC/TT | 258/28 | 85/8 | 1.15 (0.50-2.62) | 0.748 | 240/29 | 94/9 | 1.27 (0.58-2.78) | 0.557 |
|  | log-additive |  |  |  | 1.00 (0.70-1.44) | 0.984 |  |  | 0.97 (0.68-1.37) | 0.863 |

SNP, single nucleotide polymorphism; OR, odds ratio; 95% CI, 95% confidence interval.

*p* values were calculated by logistic regression analysis with adjustments for age and gender.
